# Limited transmission of cervid prions to nonhuman primates provides insights into the zoonotic potential of chronic wasting disease

**DOI:** 10.1126/sciadv.aeb7613

**Published:** 2026-05-27

**Authors:** Samia Hannaoui, Sandra Pritzkow, Wiebke M. Jürgens-Wemheuer, Dirk Motzkus, Joo-Hee Wälzlein, Karla A. Schwenke, Yo-Ching Cheng, Hanaa Ahmed Hassan, Irina Zemlyankina, Kylee Drever, Michael Beekes, Walter J. Schulz-Schaeffer, Christiane Stahl-Hennig, Sabine Gilch, Claudio Soto, Stefanie Czub, Hermann M. Schätzl

**Affiliations:** ^1^ Calgary Prion Research Unit, Faculty of Veterinary Medicine, University of Calgary, 3280 Hospital Dr NW, T2N 4Z6 Calgary, Canada.; ^2^ Hotchkiss Brain Institute and Snyder Institute for Chronic Diseases, University of Calgary, 3330 Hospital Drive NW, T2N 4N1 Calgary, Canada.; ^3^ Mitchell Center for Alzheimer’s Disease and related Brain Disorders, University of Texas McGovern Medical School at Houston, 6431 Fannin Street, Houston, TX 77030, USA.; ^4^ Institute of Neuropathology, Faculty of Medicine, Saarland University, Homburg, Germany.; ^5^ German Primate Center (DPZ), Leibniz Institute for Primate Research. Unit of Infection Models, Kellnerweg 4, D-37077 Goettingen, Germany.; ^6^ Prion and Prionoid Research Unit, ZBS 6-Proteomics and Spectroscopy, ZBS-Centre for Biological Threats and Special Pathogens, Robert Koch Institute, Nordufer 20, 13353 Berlin, Germany.

## Abstract

Chronic wasting disease (CWD) is an expanding prion disease of cervids. CWD prions persist in the environment, are shed in excreta, and accumulate in tissues of infected cervids, raising concerns about its zoonotic potential. Using cynomolgus macaques, we explored the zoonotic potential of CWD. Although most inoculated macaques remained asymptomatic, sensitive in vitro prion amplification assays revealed low levels of prions in macaque tissues. Inoculation of transgenic mice and bank voles with macaque tissues induced prion disease, achieving 100% transmission rates upon serial passage. One interpretation of these findings is that CWD prions retain infectivity across species and that primate infection may manifest atypically while still enabling transmission. Our results challenge earlier conclusions that minimize the zoonotic risk of CWD and underscore the need for continued surveillance.

## Teaser

Transmission of chronic wasting disease prions to macaques provides insights into the potential of CWD to infect humans.

## INTRODUCTION

Prions are the infectious agents causing fatal neurodegenerative diseases, including Creutzfeldt-Jakob disease (CJD) in humans, scrapie in sheep, bovine spongiform encephalopathy (BSE) in cattle, and chronic wasting disease (CWD) in cervids (*1*). These diseases are caused by the misfolding of the normal cellular prion protein (PrP^C^) into its infectious counterpart (PrP^Sc^), whose transmission across species is influenced by the prion transmission barrier, governed by PrP sequence compatibility and characteristics of the original isolate. CWD has been reported across 36 US states; 5 Canadian provinces; and in Norway, Sweden, and Finland (*2*). Affected species include white-tailed deer (WTD), mule deer, elk, red deer, moose, and reindeer (*2*, *3*). The environmental persistence of CWD, its shedding in bodily fluids and excreta, and accumulation in extraneural tissues contribute to its widespread transmission, raising concerns about zoonotic potential, particularly through venison consumption (*2*, *4*).

While BSE has demonstrated zoonotic transmission to humans [resulting in variant CJD (vCJD), (*5*)], epidemiological studies in CWD-endemic regions have shown no increased incidence of CJD or unusual human prion diseases (*6*–*9*). Numerous in vivo rodent studies indicate that the risk of CWD transmission to humans is low (*10*–*16*). However, the evolving nature and the existence of multiple CWD strains suggest the potential risk of prion adaptation to new hosts (*17*, *18*). In vitro studies using a novel human brain organoid model point at a high level of transmission barrier to humans (*19*). Other in vitro approaches involving protein misfolding cyclic amplification (PMCA), however, demonstrate that CWD can induce the pathogenic conversion of human PrP following prion strain adaptation (*20*–*22*) or directly, resulting in generation of prions transmissible to transgenic mice overexpressing human prion protein (PrP) (*23*, *24*).

In our previous study, 80% of humanized transgenic mice inoculated with CWD isolates exhibited prion seeding activity in the brain (*25*). Some showed an atypical protease-resistant PrP pattern resembling Gerstmann-Sträussler-Scheinker (GSS) syndrome and were transmissible upon second passage. These mice harbored infectious prions in feces that caused prion disease in bank voles. Together, these and other findings (*20*–*25*) suggest that the CWD-to-human transmission barrier may be less robust than previously assumed.

Nonhuman primates (NHPs) are considered relevant models for studying the risk of zoonotic prion transmission (*26*). Early studies by Lasmezas *et al.* (*27*) found histological similarities between BSE-infected macaques and patients with vCJD, confirming the link between BSE and vCJD in humans. Squirrel monkeys are susceptible to multiple prion agents, including kuru, vCJD, GSS, BSE, and CWD, whether inoculated intracerebrally or orally (*27*–*31*). However, a previous transmission study with cynomolgus macaques (*Macaca fascicularis*) has consistently led to the conclusion that CWD exhibits, at most, a very low zoonotic potential (*15*, *30*–*33*).

Our study provides evidence that cynomolgus macaques inoculated with tissues from CWD-positive cervids can accumulate detectable prion seeding activity and harbor infectious prions capable of transmitting clinical disease to CWD-susceptible rodent models. Macaque-derived central nervous system (CNS) and peripheral tissues harbored seeding-competent prions revealed by PMCA and real-time quaking-induced conversion (RT-QuIC) assays. Inoculation into transgenic mice overexpressing elk PrP^C^ induced clinical disease, albeit with low attack rates, low levels of prion seeding activity, and undetectable protease-resistant pathological PrP (PrP^Sc^). Subsequent transmission to bank voles resulted in a 100% attack rate, with a typical PrP^Sc^ signature in the brain, spinal cord, and gastrointestinal tissues. Second passage in bank voles resulted in shortened survival times, consistent with prion adaptation. Prions in gastrointestinal tissues also proved infectious and transmissible in bank voles. Passage of bank vole brain homogenates into mice overexpressing deer PrP^C^ led to a 100% transmission rate. These findings suggest that cynomolgus macaques, often considered the most relevant model for assessing prion zoonotic potential, can support CWD infection under experimental conditions, although disease manifestation is atypical and only became evident through transmission to highly susceptible rodent models with unusual tissue tropism.

While these results do not demonstrate efficient transmission of CWD to macaques, they indicate that this cross-species transmission cannot be excluded and warrants continued attention. These observations provide critical insights into how prion disease might present in the event of a spillover, reinforcing the need for sustained vigilance and further research into prion adaptation and zoonotic potential.

## RESULTS

### Transmission and pathogenic assessment of CWD in cynomolgus macaques

In this study, a cohort of 18 female cynomolgus macaques was inoculated with different CWD preparations, which included tissues from elk, mule deer, and WTD, originating from diverse geographic regions in the United States and Canada (table S1). In addition, three macaques were inoculated with material from cervids that tested negative for CWD, serving as mock controls. Animals were euthanized at predetermined time points between 4- and 10 years postinoculation.

In this study, we present the analysis of seven of the early euthanized macaques, alongside the negative controls, up to 7.5 years postinoculation. While most of the macaques remained asymptomatic in that time frame, two exhibited several clinical signs indicative of prion disease (orally inoculated macaque AU501 and intracerebrally inoculated macaque AU389), including ataxia, tremor, and anxiety (table S1). Wasting was observed in five macaques, with three cases possibly linked to diabetes, a condition common in female cynomolgus macaques of Mauritian origin. Two orally inoculated macaques, AU467 and AU501, exhibited wasting without diabetes, and one, AU501, showed CWD-related neurological signs, including anxiety, ataxia, tremor, reduced appetite, atypical vocalizations, and crouching (table S1). Body weight fluctuated in CWD-inoculated macaques, alternating between loss and gain, while the negative control macaque, AU242, displayed a stable weight trajectory until natural aging (fig. S1). Because euthanasia was predetermined, most macaques were euthanized during a period that likely corresponds to an early phase of prion disease progression.

Standard biochemical methods, i.e., immunoblotting after proteinase K (PK) digestion failed to show protease-resistant PrP^Sc^ in the CNS tissues of CWD-challenged macaques, regardless of the inoculation route. Similarly, results from histological and immunohistochemical analysis of brain and spinal cord tissues were not conclusive. Given that PrP^Sc^ may be present in quantities too low for conventional immunodetection techniques, we used two highly sensitive in vitro conversion assays to detect abnormal PrP in the CNS and spleen tissues of CWD-infected macaques.

### Ultrasensitive detection of CWD-macaque prions

We performed highly sensitive PMCA assays (*34*) to investigate whether tissues from CWD-infected macaques could seed conversion of PrP^C^ into PrP^Sc^. Brain homogenates from naïve transgenic mice overexpressing human PrP^C^ with methionine at position 129 were used as substrates, and reactions were performed in triplicate in three rounds of amplification. Our analysis included brain and spleen samples from macaques infected with CWD via different routes: orally inoculated (AU501 and AU467) and intracerebrally inoculated via steel-wire implantation (AU519 and AU389). Among these, AU501 and AU389 exhibited signs indicative of prion disease, while AU519 did not show any signs at the time of prescheduled euthanasia. In addition, we included eight negative controls from noninoculated macaques (DPZ16825, DPZ16828, CovA, CovB, CovC, CovE, CovF, and CovG) and a positive control from a BSE-infected macaque [A4; (*35*)].

**Fig. 1.**
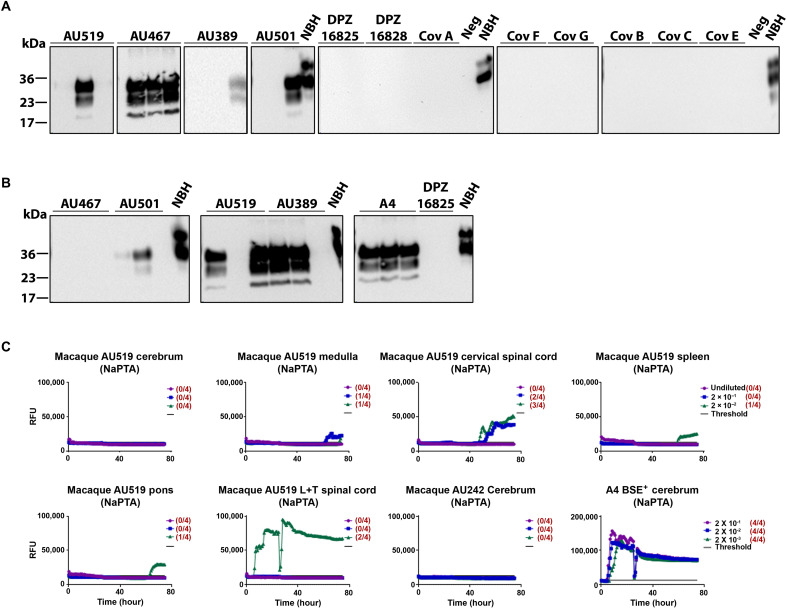
Detection of prion amplification in brain-derived and spleen samples by in vitro conversion assays. Using PMCA, (**A**) brain homogenates (BHs) or (**B**) spleen homogenates from macaques infected with CWD (AU519, AU467, AU389, and AU501), BSE (A4), and controls (DPZ 16825, DPZ16828, CovA, CovF, CovG, CovB, CovC, and CovE) were analyzed by PMCA and Western blot for the presence of PK-resistant PrP. For each sample, a 10^−2^ dilution of macaque brain or spleen homogenate was analyzed in triplicates in TgHu129M naïve BH PMCA. Three rounds of PMCA at 144, 96, and 96 cycles were conducted. The Western blot shows the PK–treated third PMCA round for all samples. The immunoblot was probed with the anti-prion–specific antibody 6D11. As a migration control, normal brain homogenate (NBH) from TgHu129M mice without PK digestion was used. Neg.: unspiked PMCA replicates of NBH. Numbers on the left indicate the position of the molecular weight markers (36, 23, and 17 kDa). (**C**) CNS tissues and spleen tissues from macaque AU519 were analyzed by RT-QuIC. The graphs depict representative RT-QuIC results after Na-PTA enrichment treatment of serially diluted (undiluted to 10^−2^) homogenates using mouse recombinant PrP (rPrP) substrate. After 25 hours of RT-QuIC reactions, the buffer was replaced with a fresh one and the assay was carried out for 50 additional hours, for a total of 75 hours. Fluorescence signals were measured every 15 min. The *x* axis represents the reaction time (hours), the *y* axis represents the relative fluorescence units (RFUs), and each curve represents a different dilution. Mean values of four replicates were used for each dilution. The cutoff (threshold) was based on the average fluorescence values of negative control + 5 × SD used in every assay.

Amplification by PMCA demonstrated a clear protease-resistant PrP^Sc^ (PrP^res^) signal in all tested brain samples from CWD-infected macaques (intracerebrally inoculated: AU519 and AU389; orally inoculated: AU501 and AU467) and the BSE-infected ones (Fig. 1A). No signal was detected in the noninfected controls (Fig. 1 and fig. S2). Notably, macaque AU467, inoculated orally, exhibited the strongest PrP^Sc^ amplification in the brain (Fig. 1A) after three rounds of PMCA, with all three replicates turning positive for PrP^Sc^, but no amplification was observed in the spleen (Fig. 1B). CWD-macaques AU501 (orally), AU519 (intracerebrally), and AU389 (intracerebrally) showed at least one positive replicate of the three in the brain and two of the three replicates positive in the spleen after PMCA at a 10^−2^ dilution of the seeds (Fig. 1, A and B, respectively).

Next, we tested serial dilutions of tissue homogenates by PMCA assay to further validate our initial findings (fig. S3). This assay successfully detected PrP^Sc^ across various dilutions and differentiated positive samples from negative controls. Except for macaque AU316 (orally), results from all tested macaques demonstrated PrP^Sc^ amplification from the CNS during the third round of PMCA, with detectable signals from a 10^−2^ dilution (intracerebrally inoculated AU519) to 10^−3^ (intracerebrally inoculated AU389 and AU469 and orally inoculated AU501) and to 10^−5^ for the intracerebrally inoculated macaque AU408. Similar to orally inoculated macaque AU467, which exhibited the strongest amplification in the brain (Fig. 1A) but no amplification in the spleen (Fig. 1B and fig. S4), macaque AU408 also showed no seeding amplification in the spleen (fig. S4). These results demonstrate the specificity of the assay, where PrP^Sc^ was detected in six of the seven brain samples, in four of the six spleen samples from CWD-inoculated macaques, and in none of the negative samples obtained from various negative macaques (see table S2).

PMCA assays of different cortex regions from orally inoculated macaque AU467 revealed varying amplification intensities (fig. S5). Cortex region IV (more caudal) exhibited the highest amplification, with all three replicates testing positive for PrP^Sc^ from the first round. In contrast, cortex region III did not show any amplification of PrP^Sc^ up to the third round. Cortex region II displayed intermediate results (more rostral), with two of the three replicates testing positive for PrP^Sc^ by the second round (fig. S5).

We used RT-QuIC as another in vitro conversion assay to assess prion seeding activity in CNS and spleen samples from CWD-inoculated macaques. The results (Fig. 1C and fig. S6) show averages of four replicates per dilution for macaques AU501 (orally) and AU519 (intracerebrally). To enhance detection sensitivity (*36*–*38*), a substrate replacement step was added after 25 hours, extending the reaction time up to 80 hours. Prion seeding activity was consistently detected in the pons and cervical spinal cord of AU501, with and without sodium phosphotungstic acid (NaPTA) enrichment treatment, and in the thoracic/lumbar spinal cord of AU519 with NaPTA enrichment. The prion seeding activity in the spleen of AU501 was positive without, but inconclusive with, NaPTA enrichment, while seeding activity in the cerebrum improved with NaPTA enrichment (fig. S6). These results confirm variation in prion seeding activity across CNS regions, supporting findings from the PMCA assay (fig. S5).

In summary, macaques infected orally (AU501 and AU467), intracerebrally (AU408 and AU469), or by intracerebrally steel wire implantation (AU519 and AU389) exhibited no detectable PrP^Sc^ signals on immunoblots and minimal prion detection via immunohistochemistry (IHC); however, prion seeding activity in brains and spleens of some animals were confirmed using highly sensitive prion conversion assays.

### Transmission of CWD-macaque prions to transgenic mice expressing cervid PrP

Subsequently, we used rodent models, including transgenic cervidized mice (TgElk) and bank voles, carrying the less susceptible methionine allele at position 109, to directly assess the infectivity and transmissibility of prions from CWD-infected macaques (CWDmac) in established bioassays. Different CNS tissues including cervical and thoracic spinal cord and caudal brain areas (medulla and pons) as well as spleen homogenates from CWD-infected macaques—AU501 and AU467 (orally) and AU519 and AU389 (intracerebrally)—were inoculated intracerebrally into TgElk mice (table S3), which are highly susceptible to CWD isolates (*39*, *40*). Mice were monitored for clinical signs of prion disease, and upon reaching experimental end points, brain, spinal cord, and spleen samples were collected.

TgElk mice inoculated with CWDmac developed prion disease at low attack rates up to 20% (one of five mice). Incubation periods ranged from 94 to 344 days postinoculation (dpi), with signs including ataxia, rigid tail, kyphosis, and weight fluctuations, similar to those observed in CWD-infected macaques. Among the tested inocula, spinal cord tissues exhibited a positivity rate of four of the seven positive mice, spleen tissues two of seven, and brain tissues a positivity of one of seven (table S3). It is noteworthy to highlight the fact that, of the four macaques tested in bioassays, three of them showed positivity in two different inocula, including two macaques inoculated orally (AU501 and AU467). However, yet again, PK–resistant PrP^Sc^ was not detectable via immunoblot in clinically and subclinically affected TgElk mice.

Using RT-QuIC, we detected low-level prion seeding in the brain, spinal cord, and spleen of TgElk mouse #2091, inoculated with cervical spinal cord material from CWDmac AU519 (intracerebrally inoculated via steel wire), terminally sick at 344 dpi (fig. S7). RT-QuIC assays confirmed prion activity in brain and spinal cord but were inconclusive in spleen samples. Similar low-level activity was detected in the brain material of other clinically affected mice, including those inoculated with samples from orally challenged macaque AU501, without or with NaPTA enrichment treatment (fig. S8, A and B, respectively), and using two recombinant PrP species, mouse and truncated hamster (fig. S8, A and B, top and bottom graphs, respectively).

Second passage in TgElk mice from spleen material of first passage mouse showed shorter survival (106 dpi), but no PrP^Sc^ was detected on immunoblot. However, RT-QuIC confirmed prion seeding activity in the spinal cord (fig. S9).

### Transmission of CWD-infected macaque prions to bank voles

**Fig. 2.**
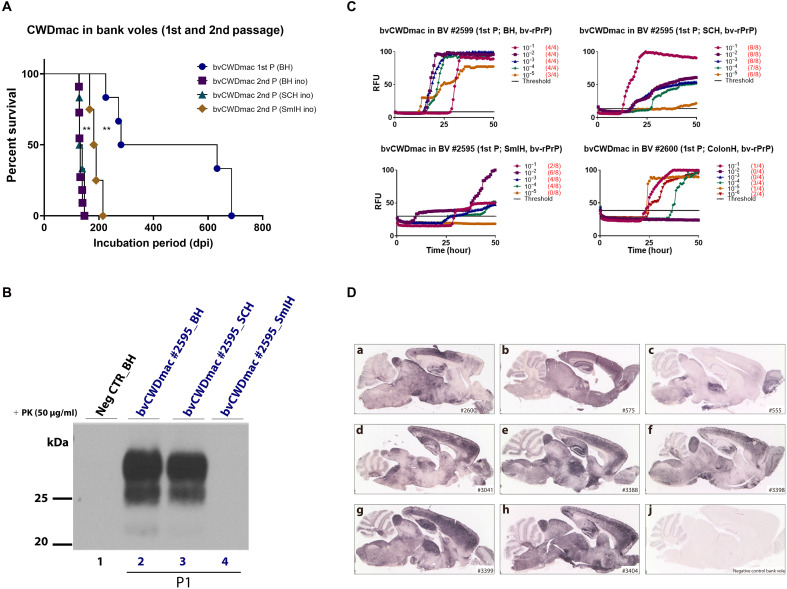
Transmission of CWD-macaque prions to bank voles. Bank voles were inoculated with second passage tgElk-CWD-macaque prions. (**A**) Kaplan-Meier survival curves of first and second passage bank voles inoculated intracerebrally. SCH, spinal cord homogenates; SmIH, small intestine homogenates. All affected voles displayed characteristic prion disease symptoms. Statistical analyses were performed using Graph-Pad software, and differences were evaluated using a log-rank (Mantel-Cox) test. **P < 0.01 [between bvCWDmac 1st P and 2nd P (BH)]. (**B**) Western blot analysis of PK-resistant PrP^Sc^ in brain, spinal cord, and small intestine homogenates from first passage bank voles. (**C**) RT-QuIC results showing prion seeding activity in brain (top left), spinal cord (top right), small intestine (bottom left), and colon (bottom right) homogenates from first passage bank voles inoculated with TgElk-CWD-macaque. The graphs depict representative RT-QuIC results of serially diluted (10^1^ to 10^5^) homogenates using bank vole (bv)–rPrP (bv-rPrP) substrate. Fluorescence signals were measured every 15 min for a total run of 50 hours. The *x* axis represents the reaction time (hours), the *y* axis represents the relative fluorescence units, and each curve represents a different dilution. Mean values of four replicates were used for each dilution. The cutoff (threshold) was based on the average fluorescence values of negative control + 5 × SD used in every assay. (**D**) PET blot analysis showing PrP^Sc^ deposits in brain tissue from first passage bank voles [(a) #2600] and second passage bank voles inoculated with brain [(d) #3041 and (e) #3388], spinal cord [(f) #3398], and small intestine [(g) #3399 and (h) #3404] materials. Two positive brains of bank voles inoculated with scrapie adapted strain RML [(b) #575] and CWD-elk [(c) #555], and an age-matched negative control (j) were also analyzed. As a primary antibody, SAF84 was used.

Bank voles are excellent hosts for various prion strains from different species, including CWD (*41*–*43*) and rare human prion diseases (*43*–*49*). Here, male and female bank voles—carrying methionine at position 109—were inoculated intracerebrally with brain and spinal cord material from first passage TgElk mouse #2091 (344 dpi) and with spinal cord homogenates from second passage TgElk mouse #2249 (fig. S10). Bank voles inoculated with spinal cord from #2249 (106 dpi) exhibited clinical prion disease with a 100% attack rate, with average survival times of 463.8 dpi (Fig. 2A). Two distinct survival clusters emerged: One group succumbed between 225 and 281 dpi, and the other succumbed between 633 and 686 dpi, irrespective of the voles’ sex.

The Western blot analysis of the brain and spinal cord revealed typical PK-resistant PrP^Sc^ with a three-band pattern, confirmed by RT-QuIC assays (Fig. 2, B and C, and fig. S11, respectively). While prion seeding was evident in the brain and spinal cord, the spleens of inoculated voles tested negative (fig. S12). This contrasts with the voles inoculated intracerebrally or intraperitoneally with original CWD isolates from deer, which showed positive prion seeding activity in the spleens (fig. S13).

We also investigated prion seeding activity in gastrointestinal tissues (small intestine and colon) from voles inoculated with spinal cord of mouse #2249. Western blot failed to detect PrP^Sc^, but RT-QuIC confirmed prion seeding activity (Fig. 2, B and C). This seeding activity in gastrointestinal tissues was specific to voles inoculated with CWD prions previously passaged into macaques and was absent in voles inoculated directly with CWD field isolates or mouse-adapted RML (Rocky Mountain Laboratory) scrapie prions (fig. S14).

**Fig. 3.**
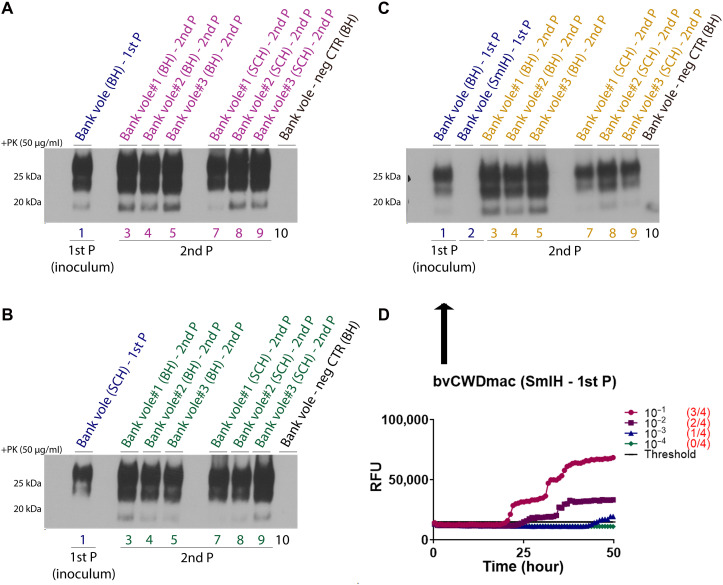
Biochemical characteristics of protease-resistant CWD-macaque prions in bank voles upon second passage. Western blot analysis of brain (lanes 3 to 5) and spinal cord (lane 7 to 9) homogenates of bank voles inoculated upon second passage with the (**A**) brain, (**B**) spinal cord, and (**C**) small intestine from first passage bank voles. Homogenates from first passage (inoculum) and second passage bank voles were digested with PK (50 μg/ml) using anti-PrP monoclonal antibody, 9A2 (amino acids 102 to 104). A negative age-matched bank vole control was also included in the Western blot. (**D**) RT-QuIC results showing positive seeding activity in the small intestine homogenates of bank vole despite the absence of PrP^Sc^ in Western blot (C, lane 2). The graphs depict representative RT-QuIC results of serially diluted (10:1 to 10:4) homogenates using bv-rPrP substrate. Fluorescence signals were measured every 15 min for a total run of 50 hours. The *x* axis represents the reaction time (hours), the *y* axis represents the relative fluorescence units, and each curve represents a different dilution. Mean values of four replicates were used for each dilution. The cutoff (threshold) was based on the average fluorescence values of negative control + 5 × SD used in every assay.

We then conducted a subsequent passage in bank voles to determine whether the prions from first passage in voles were transmissible in the same host and would adapt to their new host. All tissues used for inoculation—the brain (Fig. 3A, first lane), spinal cord [Fig. 3B, first lane), and small intestine (Fig. 3, C (second lane) and D]—induced clinical prion disease in animals with a 100% attack rate and significantly shorter survival times, indicating prion adaptation in voles (Fig. 2A). The immunoblot analysis of brain and spinal cord tissues from second-passage animals showed the presence of PK-resistant PrP^Sc^ (Fig. 3, A to C, lanes 3 to 5 and 7 to 9, respectively). Paraffin-embedded tissue (PET)–blot (PET-blot) (*50*) analyses—from first passage bank voles [(a) #2600] and second passage bank voles and inoculated with brain [(d) #3041 and (e) #3388], spinal cord [(f) #3398), and small intestine [(g) #3399 and (h) #3404]—revealed widespread complex PrP^Sc^ deposits across most brain regions (Fig. 2D), classified as type 2 prion aggregates [fig. S15 and (*51*)], with pronounced vacuolation in areas such as the frontal cortex, hippocampus, and cerebellum (fig. S16). In IHC, PrP^Sc^ deposits were found with different intensities in various regions of the brain, notably, frontal and parietal cortex, and showed plaque-like deposits associated with intense vacuolation (fig. S17). In the white matter of the cerebellum, abnormal PrP presented as coarse deposits of different sizes (fig. S17).

In parallel, we passaged brain material from TgElk mouse #2091 (344 dpi) inoculated with cervical spinal cord from CWD intracerebrally infected macaque AU519 into bank voles (see scheme fig. S10). All voles developed clinical prion disease signs with most succumbing after 800 dpi, except vole #3022, which was euthanized at 531 dpi with ataxia. Immunoblot analysis was negative for PrP^Sc^, but RT-QuIC assay confirmed prion seeding activity in brain and spinal cord tissues of vole #3022 (fig. S18). Other voles showed clinical prion signs including ataxia, severe gait abnormalities, progressive weakness, and notable weight loss and were positive for prion seeding activity in their spinal cord tissues (fig. S19).

In addition, we inoculated bank voles with spinal cord material from first passage TgElk mouse #2091 inoculated with cervical spinal cord from CWD-infected macaque AU519 (intracerebrally inoculated). From this cohort, bank vole #2583 developed terminal prion disease by 570 dpi, with prion clinical signs such as ataxia, kyphosis, and weight loss. Although immunoblot analysis of CNS material did not detect PrP^Sc^, immunohistochemical examination revealed widespread PrP deposits across all brain regions (fig. S20, top panels). Notably, there were coarse deposits (resembling plaques) observed in the parietal cortex, hypothalamus, midbrain, and the cerebellum’s granular layer (fig. S20, top panels). Concurrently, hematoxylin and eosin (H&E) staining in these areas revealed a substantial vacuolation profile, characterized by the presence of vacuoles throughout all brain regions, in stark contrast to the unaffected, age-matched bank vole control (fig. S20, bottom panels).

Together, these findings reveal that TgElk mice inoculated with macaque-passaged prions harbored infectious prions, which were successfully transmitted to bank voles with 100% attack rate and full-blown prion disease, an outcome of more adapted prions, resultant from subsequent passages in TgElk model. Consequently, these prions exhibited substantially altered strain characteristics and new tissue tropism.

### Transmission of bvCWDmac back to cervidized mice

**Fig. 4.**
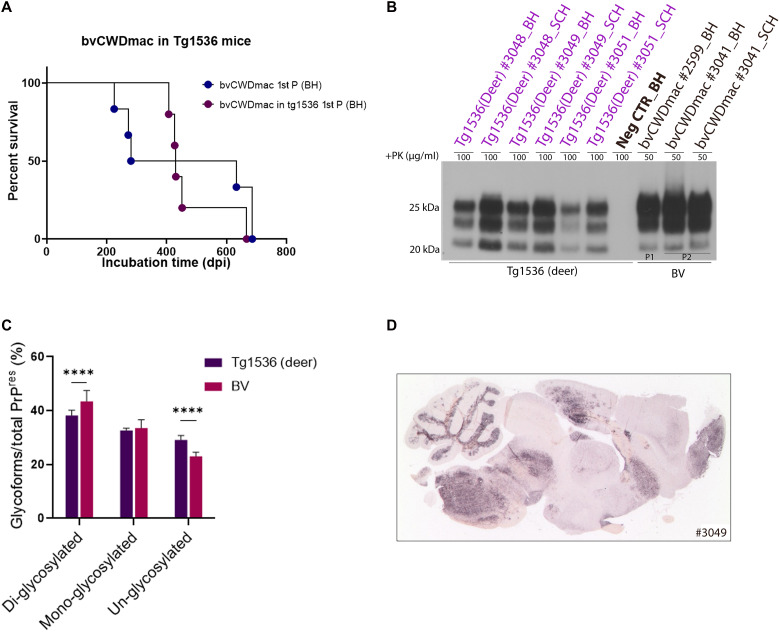
Transmission of CWD prions in TgDeer mice following inoculation with bvCWDmac material. (**A**) Kaplan-Meier curve depicting the survival times of TgDeer(1536+/+) mice inoculated with bvCWDmac (purple circles). Statistical differences were evaluated using a log-rank (Mantel-Cox) test. (**B**) Immunoblot analysis of PK-resistant PrP^Sc^ in brain and spinal cord of TgDeer and bank vole first and second passage tissues. (**C**) Quantification of the glyco-form ratio of TgDeer mice and bank voles. Statistical analyses were performed using two-way analysis of variance (ANOVA) (Tukey’s multiple comparisons test; ****P < 0.0001, means ± SD), *n* ≥ 3 biological samples. (**D**) PET-blot analysis of PrP^Sc^ deposits in TgDeer mouse brains using SAF84 anti-prion monoclonal antibody.

In the next step, we used TgDeer(1536^+/+^) mice (*52*), which overexpress deer PrP^C^, in subpassage experiments with material from bank voles (bvCWDmac) to test whether bvCWDmac could be retropassaged into a cervidized host. Transmission to TgDeer mice resulted in a 100% infection rate, with an average incubation period of 476.8 dpi. Four mice succumbed between 407 and 452 dpi, while one had a delayed onset and was terminal at 667 dpi (Fig. 4A). Immunoblot analysis showed differences in PK-resistant PrP^Sc^ between bank voles and TgDeer mice, particularly in glycoform ratios (Fig. 4, B and C). In TgDeer mice, PrP^Sc^ was also more pronounced in the spinal cord compared to the brain, contrasting with inoculated bank voles, where a notably larger volume in immunoblot was required for PrP^Sc^ detection in spinal cord (Figs. 2B, 3, A to C, and 4B). The PET-blot analysis revealed distinct PrP^Sc^ distributions, where PrP deposits were characterized by a synaptic pattern, classified as type 1 deposits in TgDeer mice, contrasting with a predominantly type 2 PrP^Sc^ deposition pattern (*53*) across all brain regions in bank voles (Figs. 2D and 4D, and fig. S15). This confirms the infectivity and transmissibility of CWDmac across species.

Together, these results underscore the potential zoonotic threat that may manifest subtly in primates while maintaining infectivity and transmissibility, opening the potential to adapt to humans. These findings prompt critical questions about the broader zoonotic landscape of CWD and emphasize the need for vigilant monitoring as CWD continues to spread through diverse ecosystems, broadening the potential for human exposure.

## DISCUSSION

The captivating history of prion diseases is deeply intertwined with the use of NHPs as experimental models. These models have provided groundbreaking insights into prion transmissibility, pathogenesis, and transmission barrier to humans (*26*). Their continued relevance lies in the unique ability of primate models to bridge experimental findings in rodents with potential implications for human health (*28*). The PrPs of cynomolgus macaques and squirrel monkeys are equally distant from human PrP (*54*). However, cynomolgus macaques are evolutionarily closer to humans, although they differ at two key residues—M166V and E168Q (*30*)—within the β2-α2 loop of PrP, a structural determinant of cross-species compatibility (*14*, *55*). Experimental studies using transgenic mouse models expressing human PrP variants have demonstrated that substitutions at these positions can modulate susceptibility to specific prion strains in a strain-dependent manner, influencing transmission efficiency and incubation time (*56*, *57*); yet, these substitutions do not negate the high overall amino acid sequence similarity, and macaques remain a suitable animal model for studying prion disease risk to humans.

Here, we report that cynomolgus macaques contain prion seeding activity after inoculation with tissues from CWD-positive cervids via both inoculation routes, oral (macaque AU501 and AU467) and intracerebral (macaque AU519, AU389, AU408 and AU469). Macaques were subjected to scheduled euthanasia, and most monkeys were euthanized between 5 and 10 years after CWD inoculation. While most macaques remained asymptomatic during these periods as these time points likely correspond to early disease stages, two developed clinical signs of prion disease, namely, wasting, ataxia, tremor, and anxiety [macaques AU389 (intracerebral route via steel wire implantation) and macaque AU501 (oral route)]. Using highly sensitive prion amplification assays (PMCA and RT-QuIC), we detected low-level prion seeding activity in brain, spinal cord, and spleen tissues of CWD-inoculated macaques, even in the absence of detectable protease-resistant PrP^Sc^ by immunoblotting. Seeding signals were observed not only at the inoculation interface but also in tissues that were downstream/distal of the inoculation site, e.g., brain and spinal cord after oral inoculation and spleen and spinal cord after intracerebral inoculation using steel wires that locally concentrate inoculating prions. This supports the interpretation that CWD prions underwent in vivo amplification rather than passive persistence of residual inoculum, an alternative explanation.

The successful transmission of CWD-macaque prions from animal AU519 (intracerebral route via steel wire implantation)—which exhibited positive prion seeding activity in both PMCA and RT-QuIC assays—first to transgenic mice overexpressing elk PrP and subsequently to bank voles provides evidence of sustained infectivity through multiple ways (fig. S10): (i) From brain tissue of CWD-macaque-infected TgElk (first passage), prion seeding activity was observed in bank voles (figs. S10, S18, and S19); (ii) from spinal cord tissue of CWD-macaque–infected TgElk, IHC confirmed PrP^Sc^ deposits, and H&E staining revealed vacuolation in bank vole #2583 (figs. S10 and S20); (iii) infectivity was further demonstrated in spleen tissue from CWD-macaque infected TgElk after adaptation within the same line (mouse #2249), with 100% attack rates observed in voles upon serial passage.

Note that prion infectivity was detected via bioassays in macaque tissues that are postsynaptic to the area where the macaques were inoculated, a finding that is difficult to reconcile with simple persistence of residual inoculum and is consistent with biological spread and possible in vivo amplification in macaques while not excluding alternative explanations. Yet, we acknowledge that persistence or dissemination of residual inoculum with subsequent selection or amplification in rodent hosts cannot be formally excluded.

Prion adaptation in voles led to a pronounced reversion to classical prion disease patterns (e.g., 100% clinical disease and detection of PK-resistant PrP^Sc^ in immunoblot). Further transmission to transgenic mice, overexpressing deer PrP (TgDeer), resulted in a 100% infection rate, highlighting the ability of CWD prions to evolve, stabilize, and crossspecies barriers. These findings suggest that while direct transmission to primates may be mostly atypical and subclinical, CWD-macaque prions retain transmissibility and infectivity. The strikingly similar phenomenon was found in foodborne BSE transmission to macaques when analyzing the challenged animals during the incubation phase. The preclinical animals showed an atypical prion pattern different from the BSE pattern but effectively transmitted the disease to reporter animals (*58*). Alternatively, our findings can be explained with instances in which prions retain their original host range properties despite undergoing structural changes in a new host species. One possible explanation aligns with the concept of nonadaptive prion amplification [NAPA; (*59*)], wherein prions replicate efficiently in a heterologous host without acquiring adaptive changes, thereby preserving the host range properties of the original strain. While this remains a hypothesis, it provides a plausible framework for interpreting our observations.

The absence of detectable PrP^Sc^ in TgElk mice following initial inoculation with CWD-macaque prions, despite clinical signs, raises intriguing possibilities. One explanation is the presence of protease-sensitive forms of PrP^Sc^, which remain infectious but are not detectable by assays targeting protease-resistant isoforms (*60*). Alternatively, the species accumulating in macaque brains may be unstable and/or represent transient toxic intermediates, yet seeding-competent PrP fragments that evade conventional detection methods but can propagate in susceptible hosts following adaptation (*61*). A relevant precedent for this exists in variably protease-sensitive prionopathy (VPSPr), in which PrP aggregates are largely protease-sensitive in humans, resulting in minimal or atypical PK-resistant bands by immunoblot, yet remain fully capable of seeding PrP substrate in PMCA and RT-QuIC (*62*) and are transmissible under specific experimental conditions, ultimately yielding a typical sporadic CJD-like biochemical signature (*47*). In VPSPr, distinct PrP^Sc^ conformers distributed across different brain regions exhibit markedly different seeding efficiencies, with the PMCA-amplified product acquiring a more classical CJD-like banding pattern despite originating from predominantly protease-sensitive conformers (*62*). These observations demonstrate that protease sensitivity does not preclude infectivity or seeding competence, and PrP^Sc^ conformers can shift into a more PK-resistant state only after amplification, whether in vitro (*62*) or in vivo (*47*). This supports the interpretation that macaque tissues containing low or undetectable PrP^Sc^ may nevertheless harbor replication-competent PrP conformers that convert into a classical PK-resistant signature only when propagated in a highly susceptible model. Further work will be required to characterize these protease-sensitive PrP conformers and define their distribution across tissues and disease stages.

After two TgElk passages, PrP^Sc^ became detectable in bank voles, suggesting that strain adaptation and conformational refinement may stabilize these intermediates into classical PrP^Sc^ forms (*63*). Alternatively, deformed templating could generate transient, toxic intermediates that drive neurodegeneration without forming stable aggregates (*63*, *64*). The observed spinal cord tropism parallels the findings from foodborne BSE transmission to macaques, which showed that the first site of entrance to the central nervous system was the lumbar spinal cord. The observed spinal cord tropism in macaques may reflect a preference for specific microenvironments or cofactors that enhance prion propagation (*65*). These findings highlight the complexity of prion adaptation and underscore the need for advanced techniques to detect subclinical prion activity.

A limitation of our study was the restricted observation period for the macaques, which were maintained for 5 to 10 years, with most undergoing scheduled euthanasia. Because of logistical constraints, it was not possible to extend the monitoring of animals to possible clinical end points. Consequently, most macaques analyzed in greater detail were at 5 to 6 years post–CWD-inoculation. In humans, incubation periods of at least 7.5 years have been documented following occupational exposure to sheep-adapted BSE prions (*66*), while cases of iatrogenic CJD and kuru have exhibited incubation times extending over several decades, up to 50 years in the case of kuru (*67*). It is therefore plausible that most of the animals in this study were euthanized during the asymptomatic, preclinical phase of disease. Supporting this interpretation, previous studies have demonstrated that PMCA can detect PrP^Sc^ signals in dilutions as high as 10^−10^ from animals or humans at the terminal stages of prion disease (*68*). In contrast, our results (fig. S3) revealed PMCA positivity only up to a 10^−5^ dilution in the strongest samples, indicating a comparatively lower, yet detectable, PrP^Sc^ seeding activity in CWD-infected macaques, consistent with what would be expected during early or preclinical disease stages. Accordingly, low PMCA amplification and the absence of terminal PrP^Sc^ pathology do not negate active prion replication when viewed in the context of preclinical prion biology in primates.

Nevertheless, our study offers pivotal insights into the zoonotic potential of CWD prions, presenting a contrast to earlier findings from other laboratories. In contrast to the findings in squirrel monkeys, where even oral inoculations transmitted CWD successfully (*29*, *30*), these studies have consistently shown no evidence of CWD prions transmission in other human models, including in the cynomolgus macaque model, showing little to no clinical disease or detectable PrP^Sc^, even after subsequent passages, suggesting very minimal or even absent zoonotic risk (*15*, *30*–*33*). However, our findings challenge this view by demonstrating that infectious prions were consistently detectable in the macaques and remained transmissible following passage in rodent models, pointing to a real zoonotic risk that may have been underestimated in earlier research studies. It is important to emphasize that our results do not imply frequent or efficient transmission to primates but instead indicate that transmission may occur at low levels and present atypically, remaining undetected by classical assays yet amplifying and retaining biological infectivity.

In our study, we exclusively used female cynomolgus macaques of a similar age range, ~4 years at the time of challenge, to ensure a controlled cohort and account for any potential age-related variations in prion susceptibility or resistance. This approach contrasts with the RML study, where both male and female macaques of varying ages were included (*31*, *32*). Furthermore, our study used a diverse array of CWD isolates, including various tissues such as muscle, and higher inoculation dosages were administered. These factors may have contributed to the increased susceptibility observed in our macaque cohort, underscoring the impact that biological and methodological differences may have on the outcomes of studies.

Nonetheless, potential alternative explanations must also be considered, e.g., the persistence of residual inoculum without replication in macaques, and possible contamination in the laboratory. The study by Béringue’s group demonstrated that infectious prions can persist in the brains of infected knockout animals for extended periods of time and remain infectious and transmissible in susceptible mouse models (*69*). In their report, the PrP knockout mice were inoculated intracerebrally, and seeding activity (RT-QuIC) and infectivity (bioassay in susceptible mice) were solely assessed in the brain—the site of infection used.

In our report, we cannot formally exclude the possibility that the infectivity detected reflects residual inoculum—it is a plausible scenario. However, the presence of infectivity in spinal cord and spleen—tissues that are postsynaptic relative to the exposure site in intracerebrally inoculated macaque AU519 (steel wire implantation)—is difficult to reconcile with persistent inoculum and instead supports trans-synaptic spread after templated amplification of host PrP in the macaques. In addition, steel wire–bound prions appear unlikely to migrate along neuroanatomical pathways without prior replication.

This interpretation aligns with previous primate transmission studies showing that preclinical BSE-exposed macaques can harbor low or undetectable classical PrP^Sc^ while retaining transmissible infectivity (*58*). More recently, Deslys, Comoy, and colleagues reported an atypical, transfusion-induced myelopathic phenotype in cynomolgus macaques that did not meet v-CJD diagnostic criteria and likewise lacked detectable CNS PrP^Sc^ despite clear evidence of infection (*70*). In these animals, spinal cord pathology was reported in the absence of detectable CNS PrP^Sc^, suggesting that neurodegeneration can occur without classical diagnostic signatures (*70*, *71*). In this context, the neurological manifestations observed in two of our macaques could possibly fall within a comparable category of atypical presentation that lacks typical prion pathology. Our bioassay data may point to a relative spinal cord and peripheral tissue tropism in CWD-infected macaques, which could help explain the emergence of neurological or systemic symptoms even when brain PrP^Sc^ is difficult to detect by conventional assays. Rather than contradicting established prion biology, such a distribution would be compatible with an early or atypical disease course in which seeding-competent PrP assemblies are present across multiple tissues but are proportionally more abundant—or more readily amplified—in the spinal cord and peripheral compartments than in the brain. This pattern would differ from the classical neuropathological phenotype typically associated with prion disease while still remaining within the spectrum of known tissue-specific and temporally dynamic prion propagation.

In our study, we used an extensive passaging history, from CWD into macaques to TgElk mice to bank voles to TgDeer mice. Initially, we transmitted CWDmac prions using the TgElk mouse line, which is highly susceptible to multiple CWD prion strains (*39*, *40*, *72*), and bank voles, known for their universal susceptibility to prion strains from various species (*43*–*49*). Despite the high susceptibility of both models to CWD prions, none of the models inoculated upon the first passage developed typical prion disease and/or showed detectable PrP^Sc^, suggesting that strain adaptation and prion replication in a new host may play critical roles in shaping the outcomes as observed in this study.

To rule out potential contamination, given the presence of other prion strains in our facility, we compared the biochemical properties of bvCWDmac to other CWD isolates and scrapie-adapted strains passaged in bank voles. Differences in glycoform ratios, nonglycosylated band migration (fig. S21), PrP^res^ distribution, and prion type in PET blots (Fig. 2D) were observed between bvCWDmac and other prion strains, for instance, bvCWD-Elk (#555) and bvME7 (#575). In addition, adjusting PK concentrations for digestion of brain homogenates and loading volumes to have comparable signals confirmed these distinctions. These findings suggest that bvCWDmac represents a prion strain originating from CWD inoculation into macaques, with a different tissue tropism, and not a result of contamination from other prion strains or residual inoculum. However, a limitation of our study is that, at the time the macaque challenges were initiated, CWD prion strains had not yet been described, and the isolates used were therefore not prion strain characterized.

Our group (*25*) and others highlighted the importance of sensitive prion detection methods on interspecies prion transmission, where RT-QuIC and PMCA proved more reliable in detecting and identifying subclinical prion infections than traditional techniques, e.g., IHC and immunoblot that showed a higher detection threshold than the amplification assays. These findings suggest that standard diagnostic tools may underestimate prion infectivity in early or subclinical stages, raising concerns about undetected transmission risks. In addition, it is worth mentioning that while several prion-positive materials were passaged into bank voles, not all successfully adapted, reinforcing the specificity of the observed results. The selective propagation of certain materials indicates a requirement for specific strain compatibility or adaptation to the bank vole model. The persistence of prion seeding activity in both symptomatic and asymptomatic animals further suggests that subclinical infections may pose a long-term zoonotic risk, especially with repeated or prolonged exposure to CWD prions, including new CWD strains with different potential to cross the species barrier to humans (*18*, *21*, *23*, *73*). Previous research has established the presence of distinct CWD prion types that result in different CWD strains in a new host (*41*, *74*, *75*), which could further complicate efforts to assess human model susceptibility to CWD. CWD prions have potential for strain adaptation/evolution in different hosts with relative resistance, suggesting that certain CWD isolates may pose a risk of crossing into new species, including intermediate hosts (*76*–*79*). Together, these observations highlight a scenario in which CWD transmission to primates may be inefficient, clinically atypical, and largely subclinical yet biologically capable of generating infectious prions with zoonotic relevance.

Previous reports demonstrated that prions can silently replicate in hosts, often going undetected by traditional diagnostic tools, while still being capable of transmission and eventual disease progression (*80*–*82*). In a previous study assessing the zoonotic potential of CWD using a transgenic humanized mouse model (*25*), we reported the involvement of gastrointestinal tissues, but we did not evaluate the transmissibility of these prions. In the present study, we demonstrated the infectivity and transmissibility of prions in gastrointestinal tissues in bank voles, where inoculation with small intestine homogenate, positive only in RT-QuIC for prion seeding activity, led to a 100% attack rate. The presence of subclinical prion carriers underscores the need for advanced diagnostic techniques to better understand prion infection’s dormant phase and to identify potential reservoirs currently overlooked in prion human diagnostics. This approach is essential to prevent transmission from asymptomatic carriers or those with atypical clinical symptoms. As the geographic range and prevalence of CWD-infected animals continue to expand, the risk of exposure to humans may escalate. As with any pathogen, an increase in CWD prevalence and prion replication provides more opportunities for adaptations, mutations, and potential zoonotic transmission; hence, our results and the growing concern for this question (*83*) warrant serious consideration.

We stress that our results do not imply efficient transmission to primates; rather, they reveal that exposure under certain conditions can lead to sustained prion infectivity that remains undetectable by classical diagnostic tools.

## MATERIALS AND METHODS

### Ethics statement

This study had the approval, and strictly followed, the guidelines of the Canadian Council for Animal Care. All experiments detailed in the study were performed in compliance with the University of Calgary Animal Care Committee under protocol number AC16-0072, AC18-0047, and AC22-0015. Before inoculation and euthanasia, isoflurane was used as anesthetic at a concentration of 5% (flow rate of 0.8 liter/min) for induction and then lowered to 0.5 to 1% for maintenance of general anesthesia during the procedure.

The cynomolgus monkeys (*M. fascicularis*) used in this report were housed at the German Primate Center (DPZ) and cared for by experienced animal caretakers according to the German Animal Welfare Act complying with the European Union guidelines on the use of NHPs for biomedical research and the Weatherall report. Animal experimentations were approved by the Lower Saxony State Office for Consumer Protection and Food Safety under the project licenses 33.11.42502-04-070/07 and 33.19-42502-04-12/0975. In agreement with §11 of the German Animal Welfare act, the German Primate Center has the permission to breed and house NHPs under the license 392001/7 granted by the local veterinary office of Göttingen. Throughout experimentation, monkeys were accommodated in groups of two per cage equipped with a perch or in single cages with constant visual, olfactory, and acoustic contact with neighboring cages if not socially compatible. In this case, small mash inserts in the cages enabled the separated monkeys to groom with their roommates. Water was provided ad libitum, and animals were fed twice a day with dry monkey biscuits containing appropriate carbohydrates, energy, fat, fiber (10%), minerals, protein, and vitamin content. Fresh fruits or vegetables with treats such as nuts, cereal pulp, and different seeds were added to the diet to make foraging more appealing. In addition, the animals received feeding puzzles, toys, and wood sticks for enrichment. During the whole study, animal caretakers monitored any signs of pain, distress, or sickness by verifying the water and food intake, feces consistency, and the general condition of the animals. In case any of the monkeys presented abnormalities, those were scored and treated by veterinarians. All animal procedures were carried out by qualified veterinarians.

### Prion material

CWD prion isolates were prepared from Canadian and US origins. CWD-elk [experimental (*84*)], WTD (experimental, farmed and hunted), and mule deer (hunted) were used to challenge cynomolgus macaques of 4 years of age, from Mauritian origin (Noveprim/Spain), with a wild-type PrP. Genomic DNA was extracted from cynomolgus macaques, and polymerase chain reaction (PCR) using primers PRPMafa129Mfor (5′-CAATCAGTGGCACAAGCCCA-3′) and PRPMafa129rev (5′-AGTACACTTGGTTGGGGTAA-3′) was performed to amplify the *PRNP* gene. PCR products were purified and sequencing confirmed codon 129 genotype to be homozygous methionine (MM). Homogenates were prepared separately as 10 or 20% (w/v) brain or muscle homogenates in phosphate-buffered saline pH 7.4 [phosphate-buffered saline (PBS)]. Aliquots were stored at −80°C until further use. At the time the macaque challenges were initiated, CWD strain characterization was not yet available; only several years later were the first studies defining distinct CWD strains reported.

### Animal study

All macaque transmissions used in this study are summarized in table S1. Initially, 18 cynomolgus macaque females were inoculated via different routes, oral and intracerebral (including steel wire implantation), and were scheduled for euthanasia at different kinetic points between ~4.5 to ~7.5 years postchallenge. In this study, because of limited funding and support, we focused on eight macaques that were euthanized earlier for further downstream analysis and transmission.

We used TgElk mice, a transgenic mouse line that overexpresses elk PrP^C^ ~2.5-fold. We intracerebrally inoculated 5 to 10 6- to 8-week-old TgElk females with 1% of different CNS and spleen materials from CWD-macaque prions as summarized in table S3. Mice were intracerebrally inoculated into the right parietal lobe using a 25-gauge disposable hypodermic needle. Strict guidelines were followed to avoid cross contamination between the CWD-macaque materials during the inoculation procedure and throughout the course of the experiment. We also conducted subpassaging in TgElk mice from first passage experiments.

Two- to 3-month-old male and female bank voles, with methionine at position 109, were intracerebrally inoculated with TgElk-CWD-macaque materials (see fig. S10). A cohort of voles was also inoculated with brain homogenate from tgElk mice infected with macaque material from AU242-negative macaque. In addition, TgDeer(PrP132M^+/+^) mouse line (*52*), overexpressing deer wild-type PrP^C^ were also used for subpassaging (see fig. S10). Inoculated mice and voles were initially monitored once a week. Upon onset of clinical signs, they were monitored daily. At terminal stage of disease, clinical mice and bank voles were exhibiting rigid tail, rough coat, lack of balance, ataxia, hunched posture, and cycles of weight loss and gain. Animals with terminal clinical signs were those with confirmatory prion signs that did progress and reached terminal stage of disease. At experimental endpoints, animals were anaesthetized and then euthanized by CO_2_ overdose. After perfusion of animals, brains were collected and either fixed in formalin or frozen at −80°C for later analysis. Some TgElk mice were euthanized upon a scheduled end point.

Attack rate was defined as the proportion of inoculated animals developing clinical signs of prion disease or testing positive for prion-related markers (e.g., PrP^Sc^ by immunoblot, IHC, or seeding assays) relative to the total number of inoculated animals.

### IHC protocol

Paraffin sections (1 to 3 μm) were placed on glass slides (SuperFrost Plus, Menzel GmbH & Co KG, Germany). After 12 hours at 56°C, the tissue was deparaffinized and rehydrated in a series of xylene and isopropanol, rinsed in distilled water, and treated with a peroxidase block containing 1% H_2_O_2_. For epitope retrieval, we immersed slides in formic acid (99%) for 30 minutes [room temperature (RT), fume hood], rinsed them with distilled water, and subjected them to treatment with citrate buffer (pH 6.1) in a steamer for 30 min. In the following, slides were rinsed with Dako wash buffer (S3006, Dako, Santa Clara, CA, USA), and the primary antibody SAF84 (A03208, Bertin Bioreagent, France) was applied 1:200 in 100 μl of Dako Real antibody diluent (S2022, Dako, Santa Clara, CA, USA) to each tissue section for 90 min (RT). After rinsing the slides with a Dako wash buffer, the secondary antibody DAKO REAL EnVision horseradish peroxidase rabbit/mouse (K5007, Santa Clara, CA, USA) was added for another 60 min (RT), and the antibody reaction was visualized using the Dako Real DAB + Chromogen (K5007, Santa Clara, CA, USA) according to the manufacturer’s instructions. Sections were lightly counterstained with hematoxylin, dehydrated, and cover-slipped with Entellan (#107960, Sigma-Aldrich, St. Louis, MA, USA).

### Paraffin-embedded tissue–blot

The PET-blot was carried out as described previously (*50*, *51*). In brief, paraffin-embedded section were cut at 1 to 3 μm, placed on nitrocellulose membranes pore size 0.45 μm (16201150, Bio-Rad, Hercules, USA), and dried for at least 2 days at 56°C before they were deparaffinized in a series of xylene and isopropanol, washed in PBS containing 0.1% Tween 20 (#3472, Caesar & Lorentz GmbH, Hilden, Germany) (PBST), and dried at RT. We used a PK (P6556, Sigma-Aldrich, St. Louis Missouri, USA) concentration of 250 μg/ml at 56°C to digest the tissue sections overnight using the pillow technique, where the prewetted membranes are placed on tissues (#8382, Kimberly-Clark professional, USA) soaked with PK buffer [tris-buffered saline with Brij (B4184, Merck KGaA, Darmstadt, Germany)] and PK. All following steps were carried out with gentle agitation at RT, and membranes were thoroughly rinsed with tris-buffered saline (pH 7.8) containing Tween 20 (TBST) between the single steps and before they were submitted to denaturation with 4 M guanidine thiocyanate (#0017.2, Carl Roth GmbH, Karlsruhe, Germany) as a first step in the morning for 15 min. Unspecific epitopes were blocked with 0.2% casein (I-BlockTM, T2015, Applied Biosystems, Foster City, CA, USA) in PBST for 45 min. The primary monoclonal antibody SAF84 (A03208, Bertin Bioreagent, France) was applied for 90 min at a dilution of 1:2000 in TBST with 0.02% casein. Following this, the membranes were incubated with a secondary goat anti-mouse alkaline phosphatase–coupled antibody (ab6790, Abcam, Cambridge, UK) at 1:2000 for a further 60 min. Rinsing with TBST was followed by rinsing with NTM buffer [100 mM NaCl, 100 mM tris (pH 9.5), and 50 mM MgCl_2_] to adjust the pH in preparation for the following formazan reaction. We mixed 4-nitroblue tetrazolium chloride (1248230500, Merck KGaA, Darmstadt, Germany) and 5 bromo-4-chloro-3-indolyl-phosphate (A1117,0001, Applichem, Darmstadt, Germany) in NTM buffer and developed the membranes under visual control without agitation, before the reaction was stopped in PBS and the sections were rinsed thoroughly in distilled water to remove excess reagents. The resulting dark purple chromogen was evaluated under a dissection microscope.

### Protease-resistant PrP^Sc^ Western blot detection

For PrP analysis, brain, spinal cord, and small intestine homogenates (20%) prepared in PBS from different animals, including a negative control, were mixed with an equal volume of 100 mM tris-HCl (pH 7.4)/2% sarkosyl for 30 min before PK (25 to 50 μg/ml; Roche) digestion for 1 hour at 37°C. The reactions were terminated by adding 1× pefabloc proteinase inhibitor (Roche). Samples were boiled in sample buffer at 100°C for 10 min. For bank vole homogenates, after protease treatment was stopped by adding 1× pefabloc, an equal volume of isopropanol/butanol (1:1 v/v) was added to the samples, which were then centrifuged at 20,000*g* for 10 min. The pellets were resuspended in denaturing sample buffer and heated for 10 min at 100°C. Fifty micrograms of protein was loaded—unless otherwise stated—separated using precast 12% NuPAGE Bis-Tris gels (Thermo Fisher Scientific), and then electrophoretically transferred to polyvinylidene difluoride (PVDF) membranes (GE Healthcare Life). PVDF membranes were blocked for 1 hour in PBS-Tween (0.1%) containing skim milk powder (5%) and probed using anti-PrP monoclonal 9A2 (Wageningen Bioveterinary Research) overnight at 4°C. Washing steps were performed using PBS-Tween buffer, followed by horseradish peroxidase–conjugated goat anti-mouse immunoglobulin G antibody (Sigma-Aldrich) for 30 min at RT. Signals were developed using ECL plus detection (Millipore). Images were acquired on x-ray films (Denville Scientific). For calculation of the glycoform ratios, ImageJ software was used to quantify and determine the relative values of PrP^res^ signals (*n* ≥ 4).

### Protein misfolding cyclic amplification

PMCA studies were done with our previously described protocols (*85*–*87*). Briefly, brain homogenates [10% (w/v)] were prepared in PBS supplemented with 1% Triton X-100, 150 mM NaCl, and cOmplete EDTA-free protease inhibitor. Debris were removed by centrifugation at 810*g* at 4°C for 1 min. As PMCA substrate, we used transgenic mice expressing human *PRNP*, with 129 M polymorphism (Tg6815 line) kindly provided by G. Telling (Colorado State University). PMCA was performed in thin PCR tubes using cycles of 20 s of sonication at an amplitude of 13 followed by 29 min and 40 s of incubation at 37°C incubator. Three rounds of PMCA were done consisting of 144, 96, and 96 PMCA cycles. The product was evaluated by Western blot after PK digestion (100 μg/ml for 1 hour at 37°C) using the 6D11 antibody.

### Preparation of recombinant PrP substrate

The mature form of mouse, truncated hamster, or bank vole (amino acids 23 to 231) PrP was cloned into pET41a expression vectors (EMD Biosciences) and expressed in *Escherichia coli* Rosetta using the Express Autoinduction System (Novagen). Inclusion bodies were prepared using the Bug Buster reagent (Novagen) and solubilized in lysis buffer [8 M guanidine-HCl (Sigma-Aldrich)], 100 mM sodium phosphate, and 10 mM tris-HCl (pH 8.0) (Sigma-Aldrich)] for 50 min at 23°C and then centrifuged at 16,000*g* for 5 min at 23°C. Binding, refolding, and elution using an AKTA Explorer system has been described previously (*36*, *37*).

### RT-QuIC assay

For brain, spinal cord, small intestine, and colon homogenates, RT-QuIC was performed as described previously (*25*). Briefly, reactions were set up in assay buffer containing 20 mM sodium phosphate (pH 6.9; Sigma-Aldrich), 300 mM NaCl (Sigma-Aldrich), 1 mM EDTA (Sigma-Aldrich), 10 μM Thioflavin T (Sigma-Aldrich), and recombinant mouse, truncated hamster, or bank vole PrP substrate (0.1 mg/ml) as stated in the figure legends. Four replicate reactions, unless otherwise stated, were seeded each time with 2 μl of serially diluted homogenates from CWD-macaque, infected mice, or bank voles. Tissue homogenates (seeds) were 10-fold serially diluted in RT-QuIC seed dilution buffer [0.05% (w/v) SDS in 1× PBS]. The plate was sealed with Nunc Amplification Tape (Nalge Nunc International) and placed in a BMG Labtech FLUOstar Omega fluorescence plate reader that was preheated to 42°C for a total of 50 hours or with an additional step of substrate replacement after 25 hours, with cycles of 1-min double orbital shaking (700 rpm) incubation and 1-min resting throughout the assay time. Thioflavin T fluorescence signals of each well were read and documented every 15 min, then the values the relative fluorescence units were plotted as the average of octuplicate reactions by using GraphPad Prism (version 10.2) software. For each assay, corresponding tissue from an age- matched noninfected animals was added as a negative control. The threshold was calculated based on the average fluorescence values of negative control +5SD.

### NaPTA precipitation

NaPTA precipitation was performed on 20% (w/v) BH prepared in PBS. For RT-QuIC, 250 μl of BH was mixed with Sarkosyl to a final concentration of 2% and incubated at 37°C with shaking for 30 min. Following Sarkosyl treatment, NaPTA stock solution [20 mM phosphotungstic acid, 400 mM MgCl_2_, and 200 mM NaOH (pH 7.4)] was added at ^1^/_12.5_ of the sample volume, and samples were incubated at 37°C with shaking for 2 hours. The samples were centrifuged at 21,000*g* for 30 min at 8°C, and the resulting pellet was washed with cell lysis buffer (10 mM tris-HCl (pH 7.5), 100 mM NaCl, 10 mM EDTA, 0.5% Triton X-100, and 0.5% sodium deoxycholate) containing 1% sarkosyl. After an additional centrifugation for 15 min, the final pellet was dissolved in RT-QuIC seed dilution buffer for RT-QuIC analysis.

### Statistical analysis

GraphPad Prism 10.2 software (GraphPad) was used to draw the RT-QuIC graphs and quantification of glycoform ratios and do the statistical analyses.
