# Supplementary material for: Limited transmission of cervid prions to nonhuman primates provides insights into the zoonotic potential of chronic wasting disease
Source: Sci Adv. 2026 May 27;12(22):eaeb7613. doi: 10.1126/sciadv.aeb7613 (PMC13215175; doi:10.1126/sciadv.aeb7613)
Supplement: Supplementary file 1 — Figs. S1 to S21 Tables S1 to S3 [file sciadv.aeb7613_sm.pdf]

Science Advances
AAAS

Supplementary Materials for

Limited transmission of cervid prions to nonhuman primates provides insights into the zoonotic potential of chronic wasting disease

Samia Hannaoui *et al.*

Corresponding author: Stefanie Czub, stefanie.czub37@gmail.com;
Hermann M. Schätzl, hschaetz@ucalgary.ca

Sci. Adv. **12**, eaeb7613 (2026)
DOI: 10.1126/sciadv.aeb7613

This PDF file includes:

Figs. S1 to S21
Tables S1 to S3

Supplementary Figures

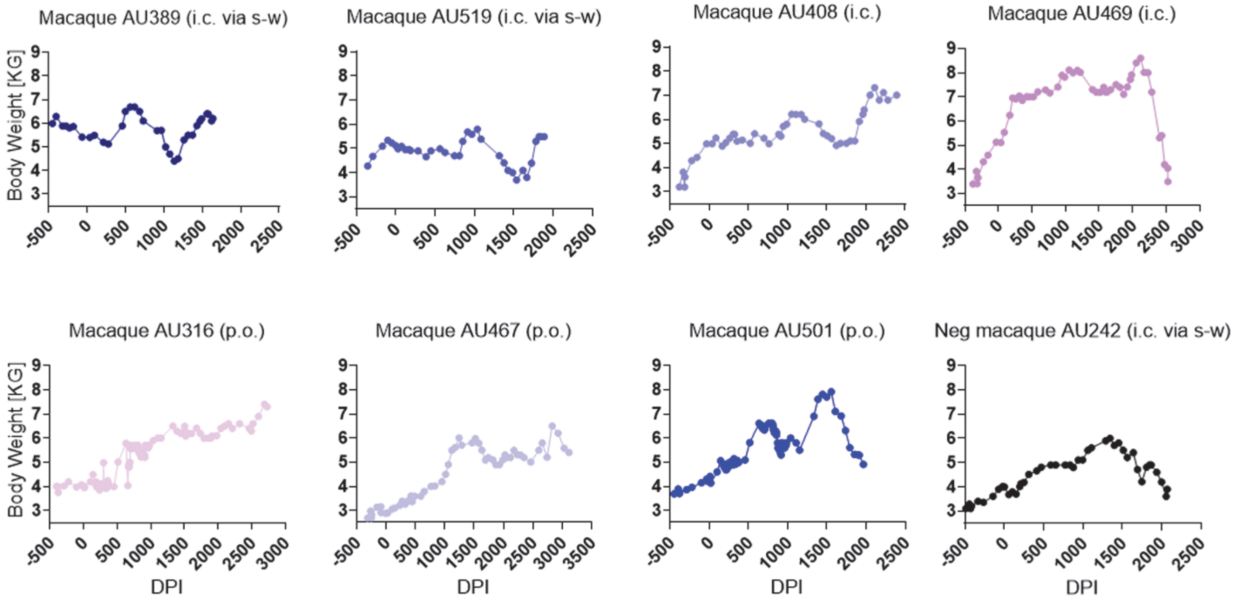

fig S1. Weight progression in macaques over time across experimental groups.
Longitudinal weight progression of macaques challenged via intracerebral (macaques AU389 and AU519 (via steel wire, s-w), 408, and 469), and oral (macaques AU316, AU467, and AU501) inoculation routes, alongside a negative control macaque inoculated intracerebrally via steel wire implantation (AU242). Data tracked from 500 days pre-inoculation through to experimental endpoint, providing a comparative overview of weight trends associated with each experimental group.

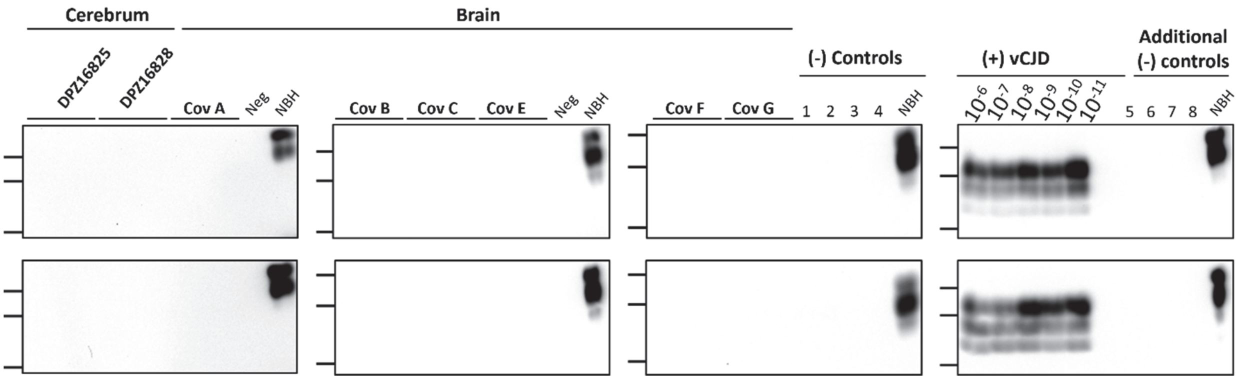

fig S2. Validation of the specificity of PMCA assay in positive and negative controls.
Brain homogenates from negative control macaques (DPZ 16825, DPZ16828, CovA, CovB, CovC, CovE, CovF and CovG), and positive control vCJD were analyzed by PMCA and Western blot for the presence of protease K resistant PrP protein. For each sample a 10^{-2} dilution of negative macaque or of vCJD brain homogenate samples was analyzed in triplicates in TgHu129M brain homogenate (BH) PMCA. Two rounds of PMCA at 144 (upper blots) and 96 (lower blots) cycles were conducted. The immunoblot was probed with the anti-prion specific antibody 6D11. As a migration control, normal brain homogenate (NBH) from TgHu129M brain homogenate without PK digestion was used. Neg.: unspiked PMCA replicates of NBH. Numbers on the left indicate the position of the molecular weight markers (36, 23 and 17 kDa).

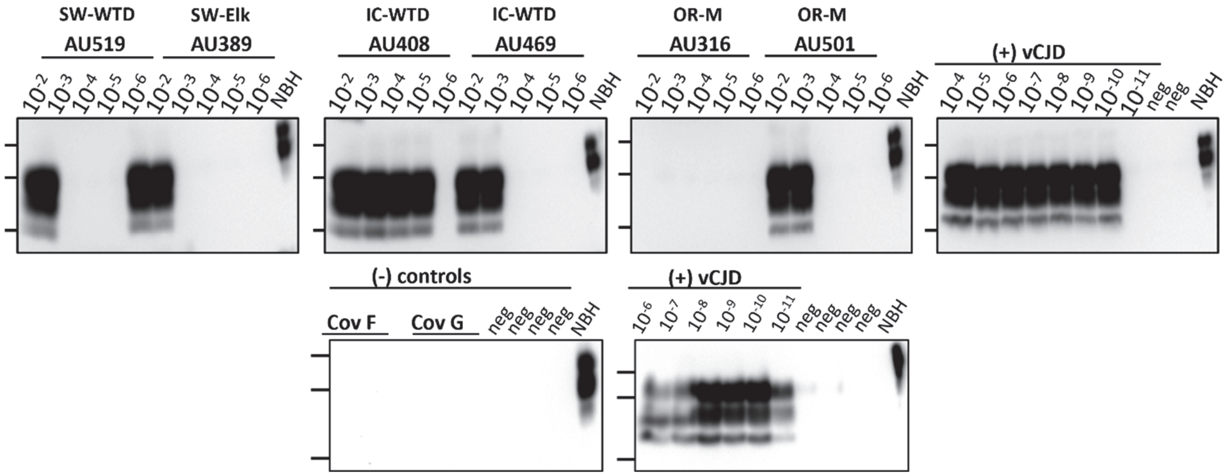

fig S3. Successful detection of prions in serial dilution PMCA.

Brain homogenates from macaques infected with CWD (AU519, AU389, AU408, AU469, AU316, and AU501), with vCJD and uninoculated negative controls (CovF and CovG) were analyzed by PMCA and Western blot for the presence of protease K resistant PrP protein. For each sample, a serial dilution from 10^{-2} to 10^{-6} dilution of brain homogenate samples was analyzed in TgHu129M brain homogenate (BH) PMCA. Three rounds of PMCA at 144, 96 and 96 cycles were conducted. The above Western blot shows the proteinase K treated third PMCA round for all samples. The immunoblot was probed with the anti-prion specific antibody 6D11. As a migration control, normal brain homogenate (NBH) from TgHu129M brain homogenate without PK digestion was used. Neg.: unspiked PMCA replicates of NBH. Numbers on the left indicate the position of the molecular weight markers (36, 23 and 17 kDa).

Different routes were used for inoculation: SW: steel wire route; IC: intracerebral route; OR: oral route. Several CWD inocula were used for inoculation: White-tailed deer (WTD), elk, mule deer (M).

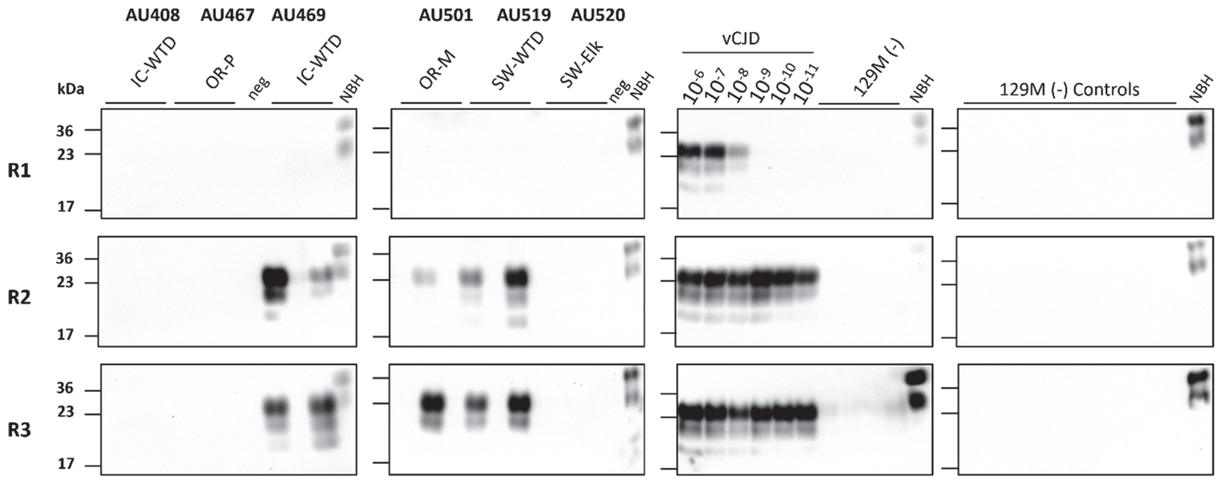

fig S4. Detection of prion amplification in spleen samples by PMCA.

Spleen homogenates from macaques infected with CWD (AU408, AU467, AU469, AU501, AU519, and AU520), vCJD and negative controls were analyzed by PMCA and Western blot for the presence of protease K resistant PrP protein. For each sample a 10^{-2} dilution of spleen homogenate samples was analyzed in triplicates in TgHu129M brain homogenate (BH) PMCA. Three rounds of PMCA at 144, 96 and 96 cycles were conducted. The above Western blots show the proteinase K treated across the three PMCA rounds for all samples. The immunoblot was probed with the anti-prion specific antibody 6D11. As a migration control, normal brain homogenate (NBH) from TgHu129M brain homogenate without PK digestion was used. Neg.: unspiked PMCA replicates of NBH. Numbers on the left indicate the position of the molecular weight markers (36, 23 and 17 kDa).

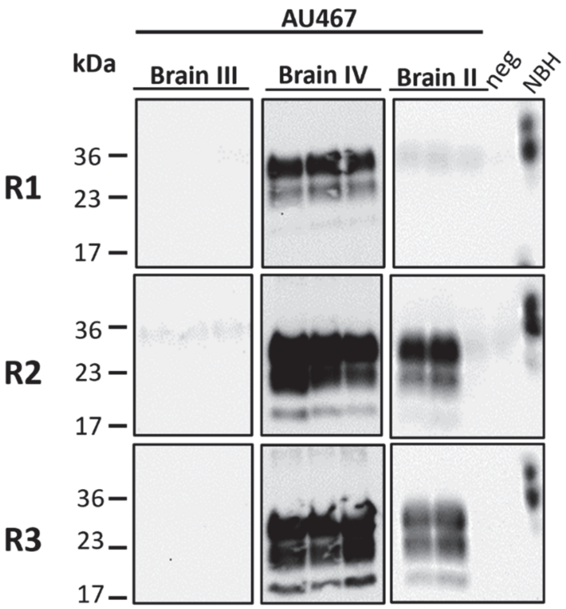

fig S5. Detection of prion amplification by PMCA depends on the load of prions in different brain regions.

Homogenates from three different brain regions (cerebrum or cortex) of macaque AU467 infected with CWD, and negative controls were analyzed by PMCA and Western blot for the presence of protease K resistant PrP protein. For each sample a 10^{-2} dilution of brain homogenate samples was analyzed in triplicates in TgHu129M brain homogenate (BH) PMCA. Three rounds of PMCA at 144, 96 and 96 cycles were conducted. The above western blots show the proteinase K treated across the three PMCA rounds for all samples. The immunoblot was probed with the anti-prion specific antibody 6D11. As a migration control, normal brain homogenate (NBH) from TgHu129M brain homogenate without PK digestion was used. Neg.: unspiked PMCA replicates of NBH. Numbers on the left indicate the position of the molecular weight markers (36, 23 and 17 kDa).

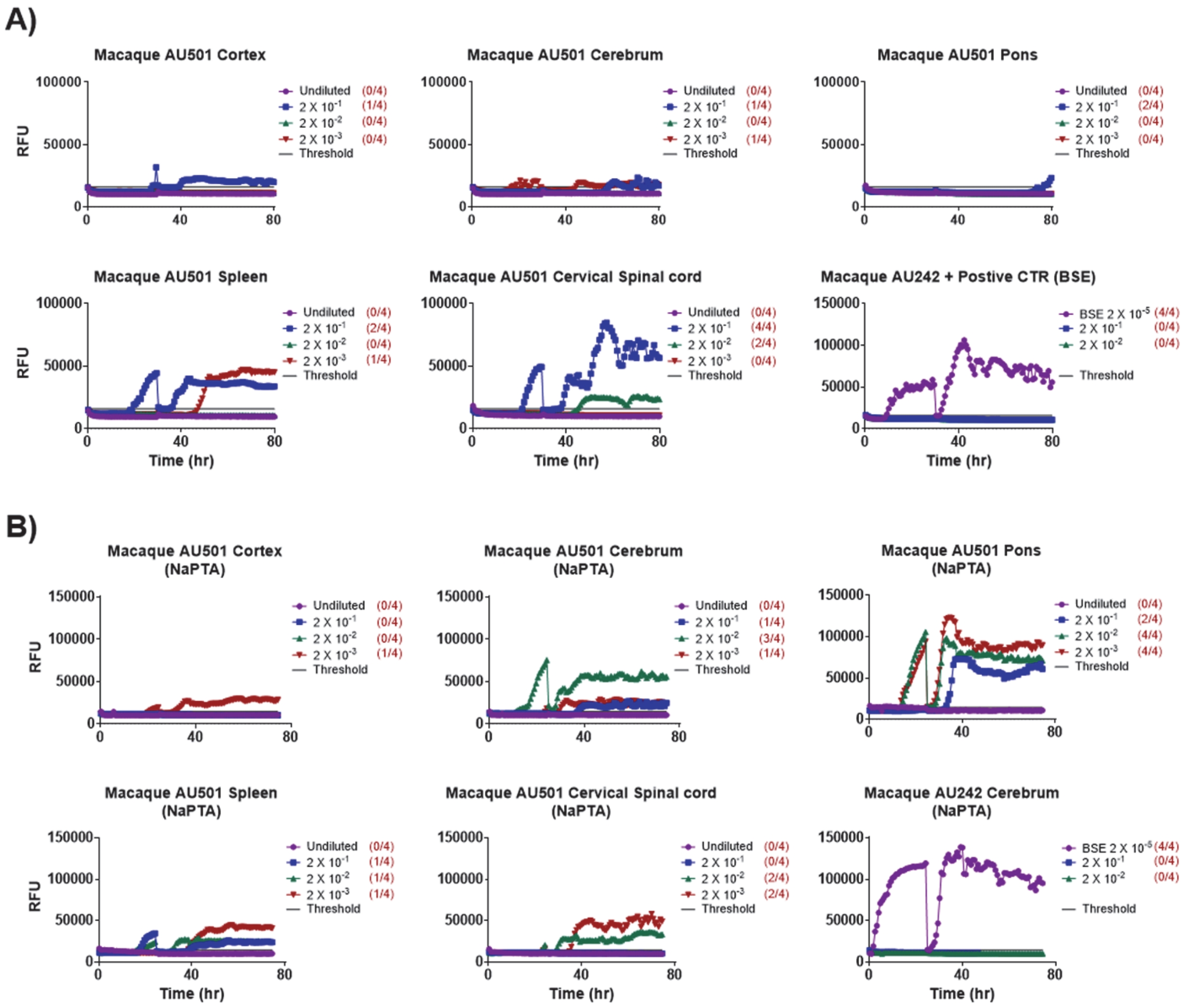

fig S6. CNS tissues and spleen tissues from orally inoculated macaque AU501, negative control, and BSE positive control were analyzed by RT-QuIC.

The graphs depict representative RT-QuIC results with A) and without B) Na-PTA enrichment treatment of serially diluted (undiluted to 10^{-3}) homogenates using mouse rPrP substrate. After 25 hours of RT-QuIC reactions, the buffer was replaced with a fresh one and the assay was carried out for 50 additional hours, for a total of 75 hours. Fluorescence signals were measured every 15 min. The *x*-axis represents the reaction time (hours), the *y*-axis represents the relative fluorescence units, and each curve represents a different dilution. Mean values of four replicates were used for each dilution. The cut-off (threshold) was based on the average fluorescence values of negative control + 5 × SD used in every assay.

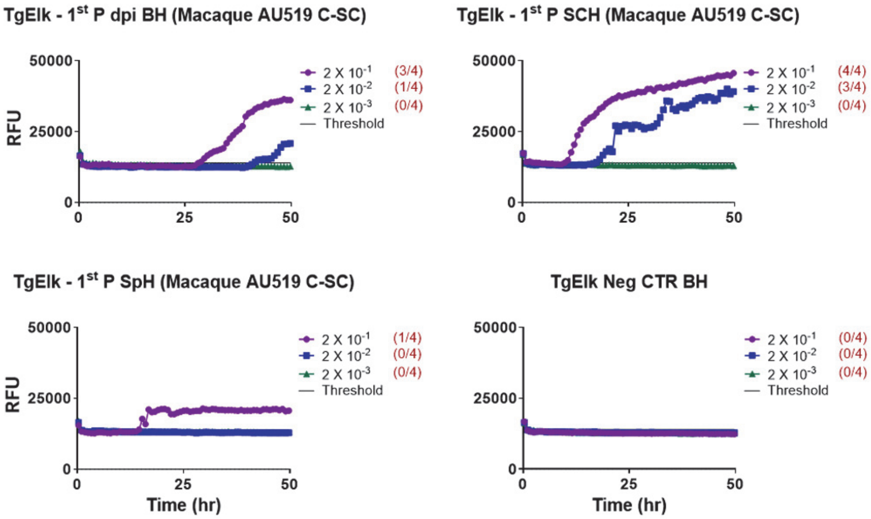

fig S7. CNS tissues and spleen tissue from CWD-macaque AU519 inoculated tgElk.
First passage TgElk mouse #2091 (344 dpi) i.c. inoculated with CWD-macaque AU519 (i.c. s-w) and negative control were analyzed by RT-QuIC. The graphs depict representative RT-QuIC results using Na-PTA enrichment treatment of brain (BH), spinal cord (SCH), and spleen (SpH) serially diluted (10^{-1} to 10^{-3}) homogenates using mouse rPrP substrate. Fluorescence signals were measured every 15 min for a total of 50 hours. The *x*-axis represents the reaction time (hours), the *y*-axis represents the relative fluorescence units, and each curve represents a different dilution. Mean values of four replicates were used for each dilution. The cut-off (threshold) was based on the average fluorescence values of negative control + 5 × SD used in every assay.

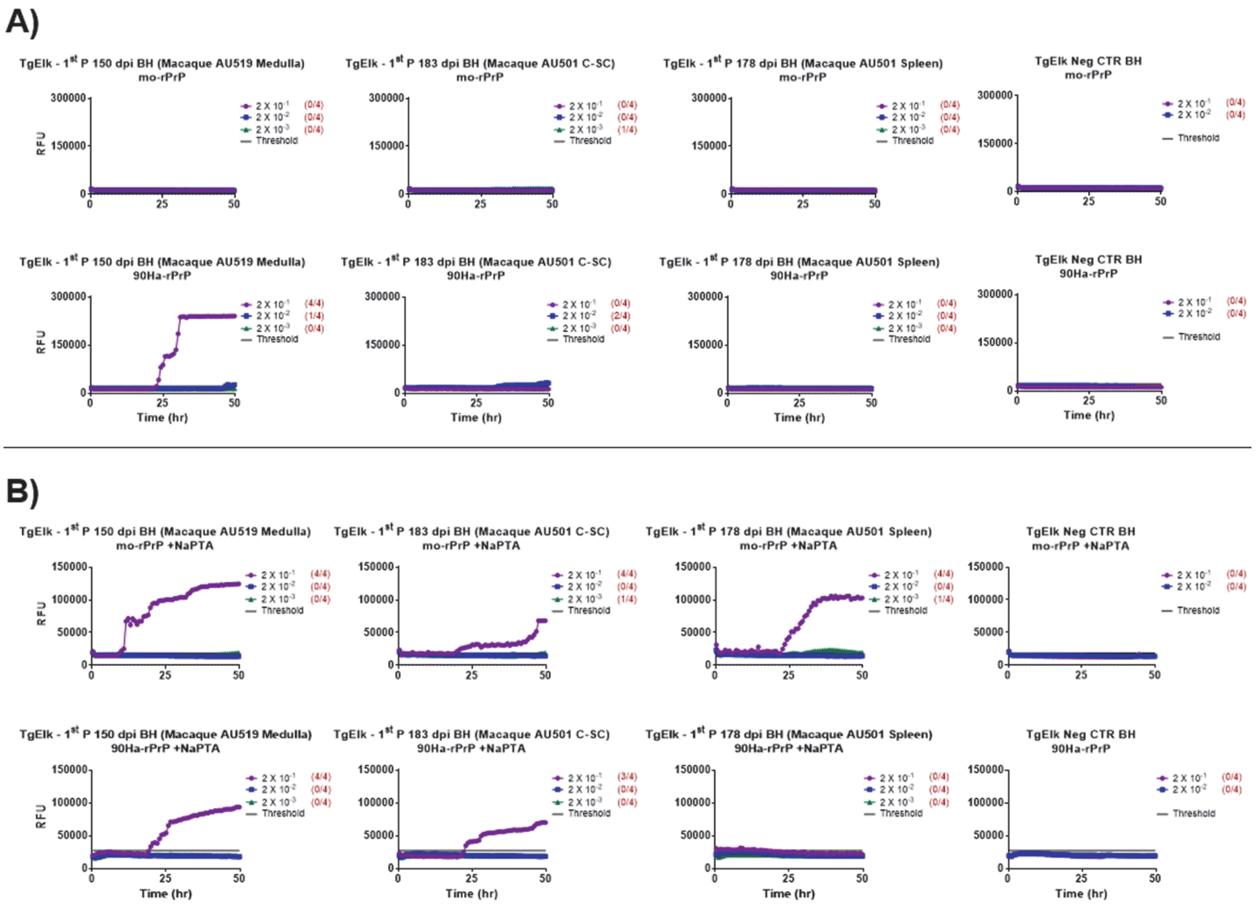

fig S8. Brain tissues of TgElk mice inoculated with CWD-macaques AU519 (i.c. s-w) and AU501 (oral).

TgElk mice were inoculated with different materials from CWD-inoculated macaques via intracerebral and oral routes and were scheduled for euthanasia at different kinetic endpoints. Brain homogenates (BH) of these mice and a negative age-matched control mouse were analyzed by RT-QuIC. The graphs depict representative RT-QuIC results without A) or with (B) Na-PTA enrichment treatment of serially diluted (10^{-1} to 10^{-3}) homogenates using mouse (mo) and truncated hamster (90Ha) rPrP substrate, upper and lower panels respectively. Fluorescence signals were measured every 15 min for a total run of 50 hours. The *x*-axis represents the reaction time (hours), the *y*-axis represents the relative fluorescence units, and each curve represents a different dilution. Mean values of four replicates were used for each dilution. The cut-off (threshold) was based on the average fluorescence values of negative control + 5 × SD used in every assay.

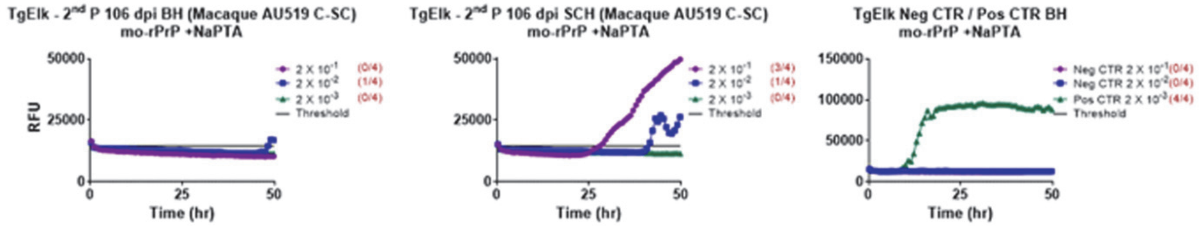

fig S9. CNS tissues of second passage TgElk mouse inoculated with CWD-macaque AU519. TgElk mouse #2249 was inoculated with spleen homogenates of TgElk mouse #2091 and euthanized upon reaching a terminal endpoint at 106 dpi. Brain homogenates (BH) and spinal cord homogenated (SCH) of this mouse and a positive and a negative age-matched control mouse were analyzed by RT-QuIC. The graphs depict representative RT-QuIC results after Na-PTA enrichment treatment of serially diluted (10^{-1} to 10^{-3}) homogenates using mouse rPrP substrate. Fluorescence signals were measured every 15 min for a total run of 50 hours. The *x*-axis represents the reaction time (hours), the *y*-axis represents the relative fluorescence units, and each curve represents a different dilution. Mean values of four replicates were used for each dilution. The cut-off (threshold) was based on the average fluorescence values of negative control + 5 × SD used in every assay.

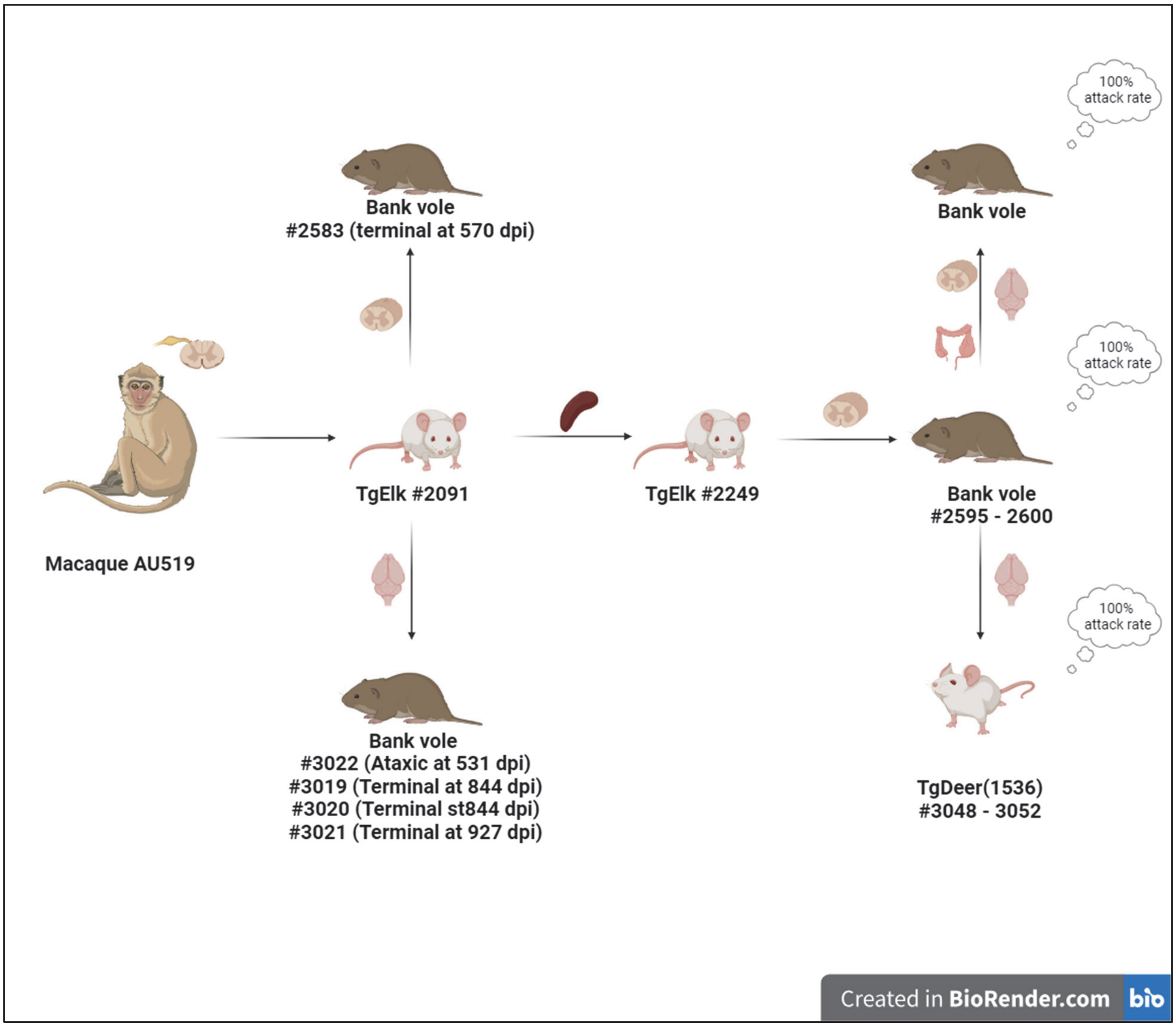

fig S10. Schematic representation of the experimental design, illustrating the passage of CWD-macaque AU519 prions in TgElk and bank vole models.

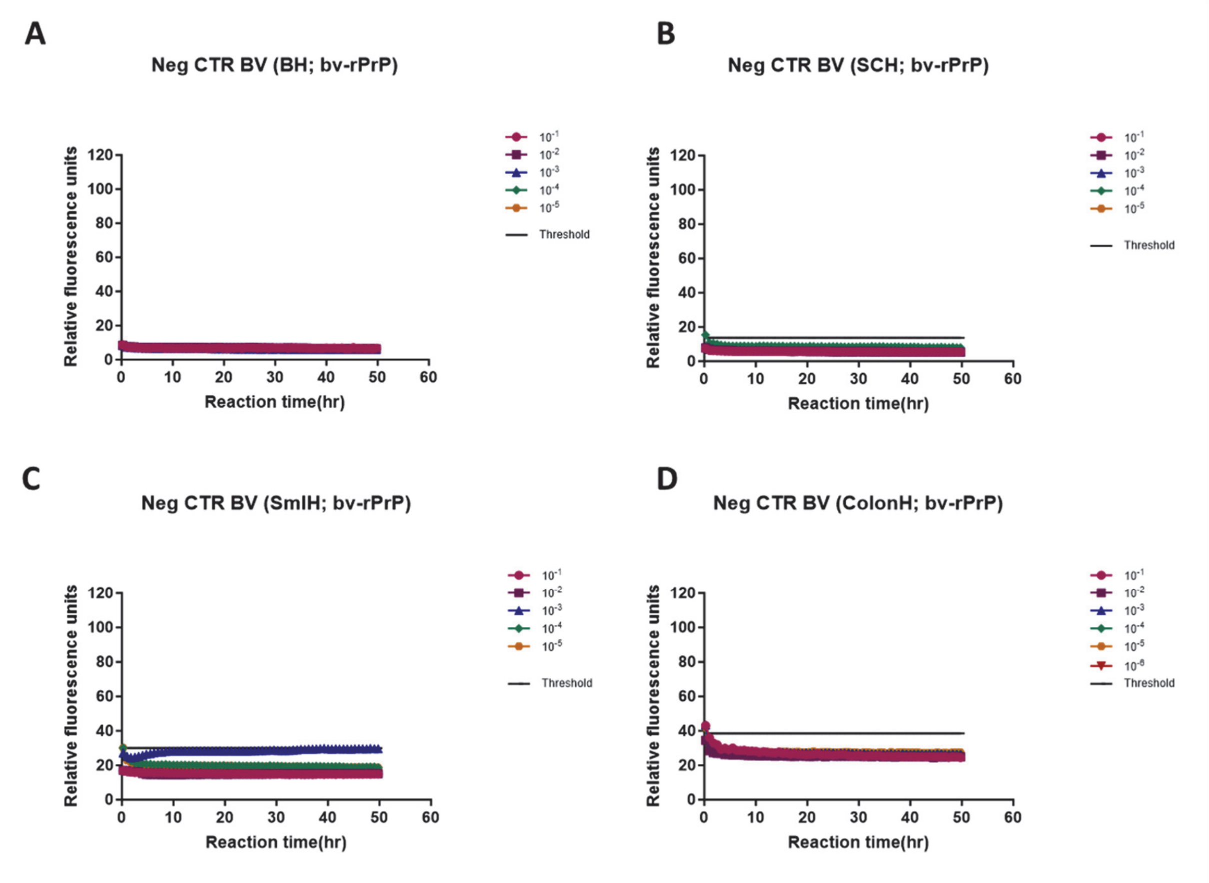

fig S11. RT-QuIC results from homogenates (brain, spinal cord, small intestine and colon) of an age-matched negative bank vole control.

The graphs depict representative RT-QuIC results of serially diluted (10^{-1} to 10^{-5}) homogenates using bank vole (bv) rPrP substrate. Fluorescence signals were measured every 15 min for a total run of 50 hours. The *x*-axis represents the reaction time (hours), the *y*-axis represents the relative fluorescence units, and each curve represents a different dilution. Mean values of four replicates were used for each dilution. The cut-off (threshold) was based on the average fluorescence values of negative control + 5 × SD used in every assay.

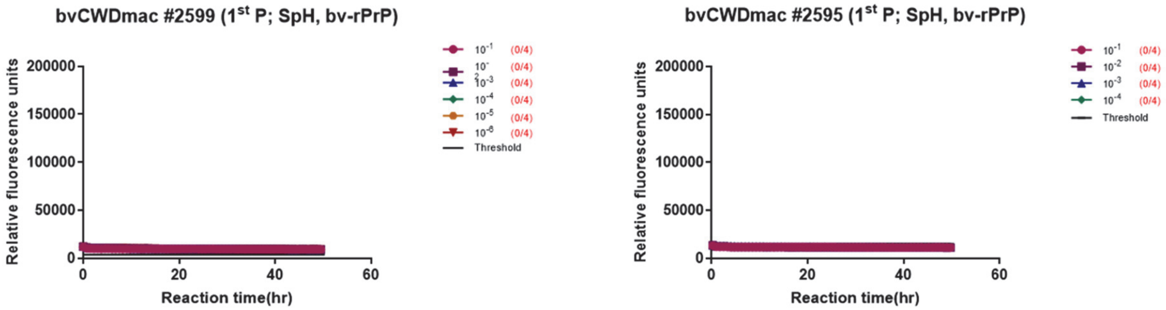

fig S12. RT-QuIC results showing negative seeding activity in spleen homogenates of first passage bank voles inoculated with TgElk-CWD-macaque prions.

The graphs depict representative RT-QuIC results of serially diluted (10^{-1} to 10^{-6} for voles #2599 and 10^{-1} to 10^{-4} for vole #2595) homogenates using bank vole (bv) rPrP substrate. Fluorescence signals were measured every 15 min for a total run of 50 hours. The *x*-axis represents the reaction time (hours), the *y*-axis represents the relative fluorescence units, and each curve represents a different dilution. Mean values of four replicates were used for each dilution. The cut-off (threshold) was based on the average fluorescence values of negative control + 5 × SD used in every assay.

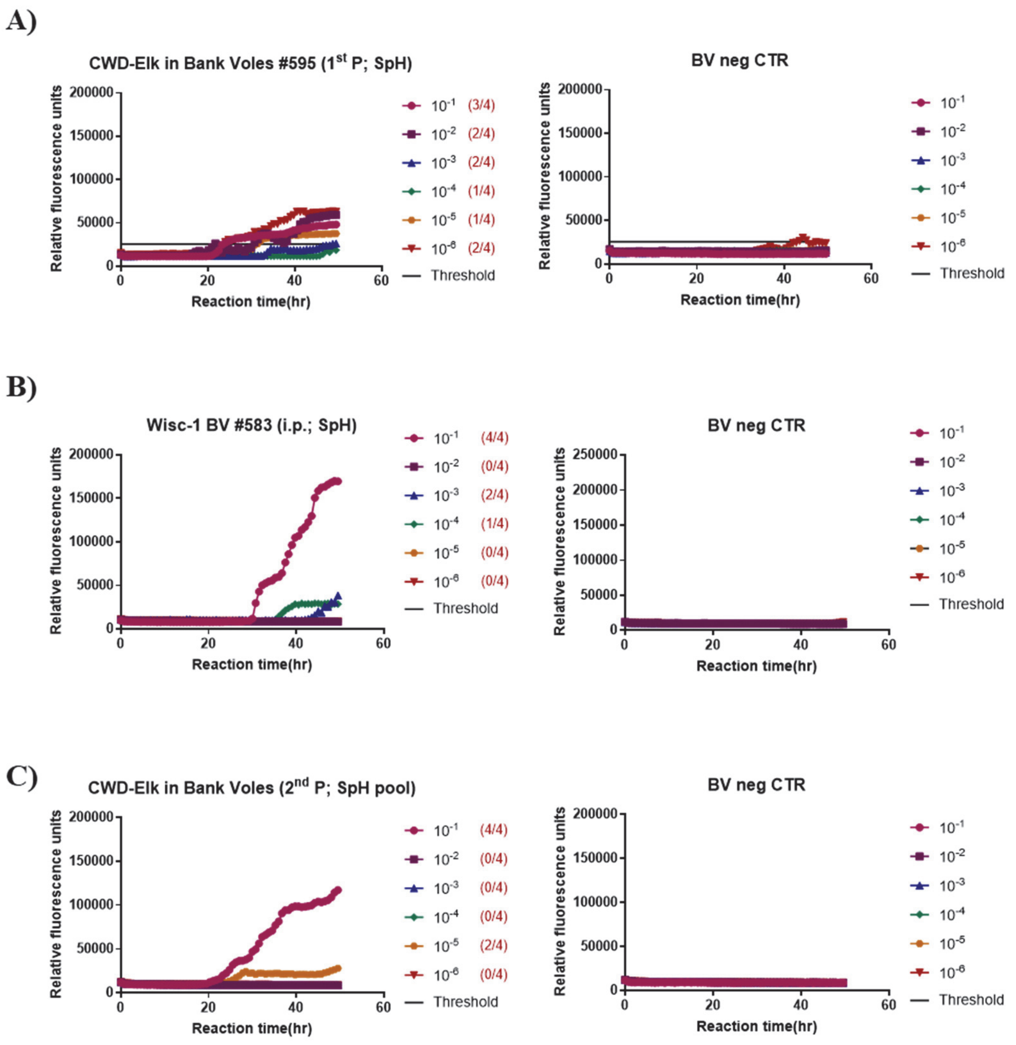

fig S13. RT-QuIC results showing positive seeding activity in spleen homogenates of bank voles inoculated i.p. A) and B) or i.c. C) with CWD-Elk and Wisc-1 prions.
The graphs depict representative RT-QuIC results of serially diluted (10^{-1} to 10^{-6}) homogenates using bank vole (bv) rPrP substrate. Fluorescence signals were measured every 15 min for a total run of 50 hours. The *x*-axis represents the reaction time (hours), the *y*-axis represents the relative fluorescence units, and each curve represents a different dilution. Mean values of four replicates were used for each dilution. The cut-off (threshold) was based on the average fluorescence values of negative control + 5 × SD used in every assay.

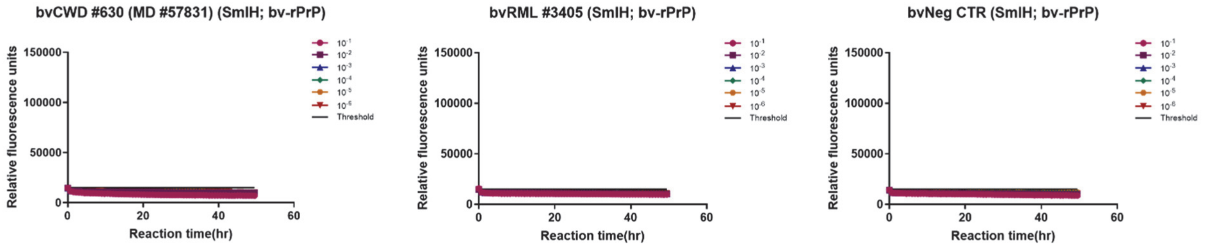

fig S14. RT-QuIC results showing negative seeding activity in small intestine homogenates of CWD-mule deer and scrapie adapted RML prion strain inoculated bank voles.
The graphs depict representative RT-QuIC results of serially diluted (10^{-1} to 10^{-6}) homogenates using bank vole (bv) rPrP substrate. Fluorescence signals were measured every 15 min for a total run of 50 hours. The *x*-axis represents the reaction time (hours), the *y*-axis represents the relative fluorescence units, and each curve represents a different dilution. Mean values of four replicates were used for each dilution. The cut-off (threshold) was based on the average fluorescence values of negative control + 5 × SD used in every assay.

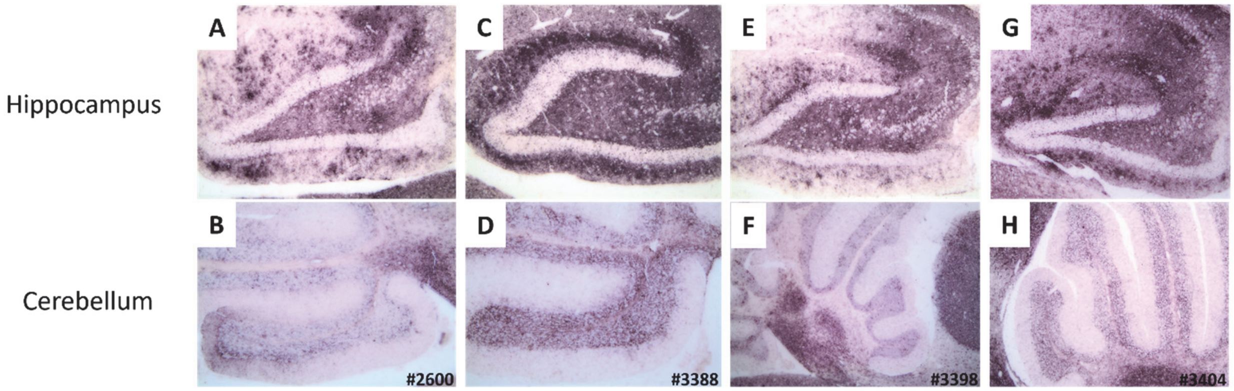

fig S15. PET blots showing widespread PrP^{res} deposits.
PET blot analyzes of hippocampus (A, C, E, and G) and cerebellum (B, D, F, and H) of first (A and B) and second passage (C-H) bank voles inoculated with TgElk-CWD-macaque AU519.

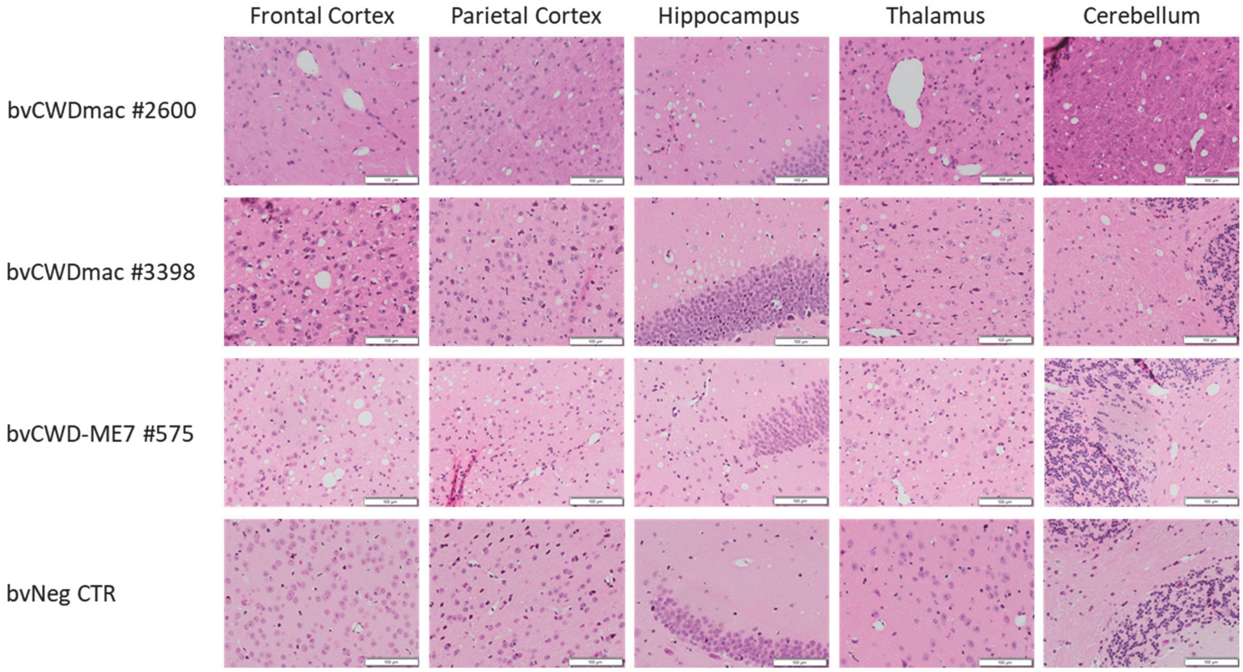

fig S 16. Hematoxylin and Eosin (H&E) staining of brain tissues of first and second passage bank voles inoculated with TgElk-CWD-macaque AU519.

Panels are stained with H&E and depicts spongiform vacuoles of first passage #2600 and second passage #3398 bank voles, alongside RML-inoculated bank vole (#575) and a negative age-matched control. Different regions of the brain are shown: frontal cortex, parietal cortex, hippocampus, thalamus, and cerebellum.

Scale bars 100 µm.

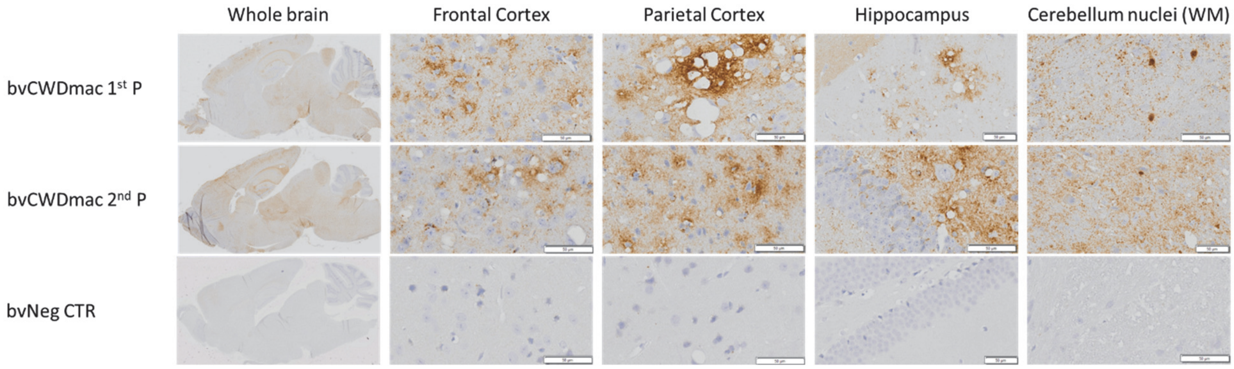

fig S17. Immunohistochemistry of brain regions from first and second passage bank voles inoculated with TgElk-CWD-macaque AU519.

Immunohistochemistry of first passage and second passage ban voles, and an age-matched negative control, using SAF84 mAb shows PrP^{Sc} deposits in the frontal and parietal cortex, hippocampus and cerebellum nuclei of the white matter (WM). Scale bars 50 µm.

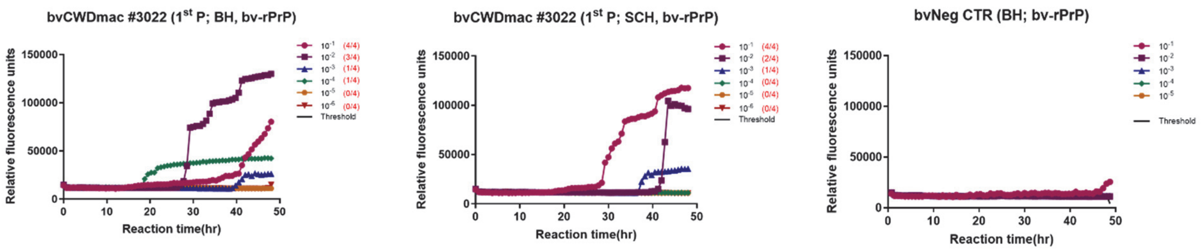

fig S18. RT-QuIC results showing prion seeding activity in brain and spinal cord tissues of bank vole #3022, inoculated with TgElk-CWD-macaque.

Bank vole #3022 was inoculated with brain homogenates of first passage TgElk-CWD macaque prions. The graphs depict representative RT-QuIC results of serially diluted (10^{-1} to 10^{-6}) brain (BH) and spinal cord (SCH) homogenates using bank vole (bv) rPrP substrate. Fluorescence signals were measured every 15 min for a total run of 50 hours. The *x*-axis represents the reaction time (hours), the *y*-axis represents the relative fluorescence units, and each curve represents a different dilution. Mean values of four replicates were used for each dilution. The cut-off (threshold) was based on the average fluorescence values of negative control + 5 × SD used in every assay.

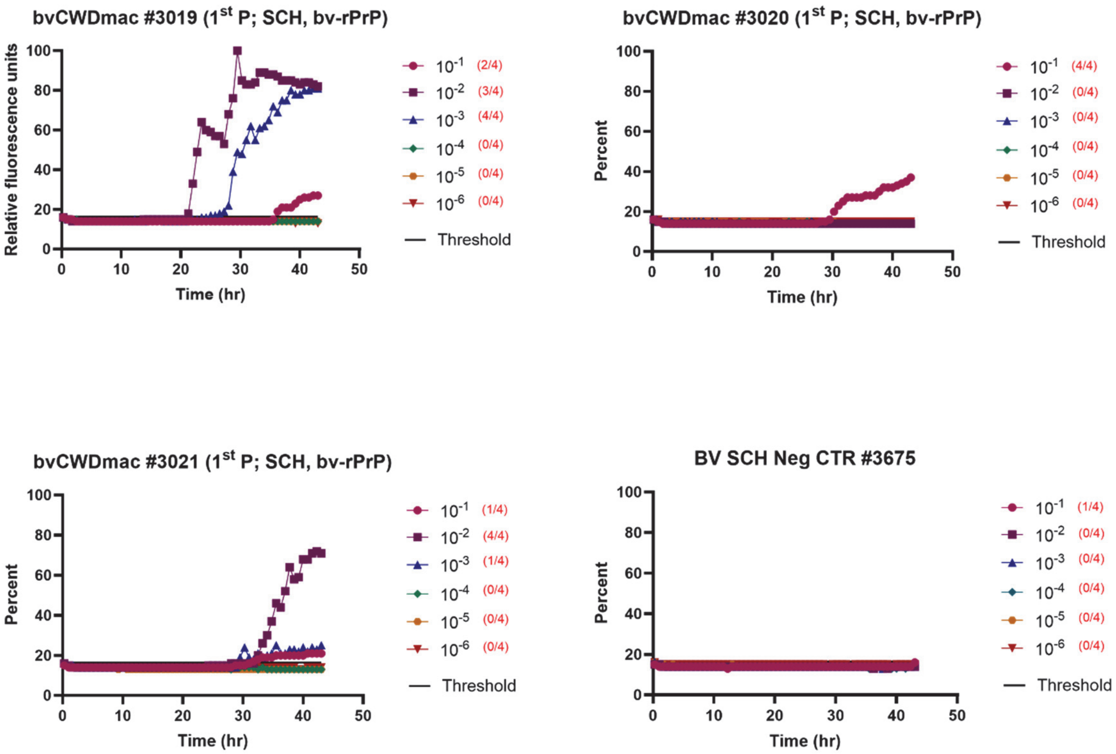

fig S19. RT-QuIC results showing prion seeding activity in spinal cord tissues of bank voles inoculated with TgElk-CWD-macaque.

Bank voles #3019, 3020, and 3021 were inoculated with brain homogenates of first passage TgElk-CWD macaque prions. The graphs depict representative RT-QuIC results of serially diluted (10^{-1} to 10^{-6}) spinal cord (SCH) homogenates using bank vole (bv) rPrP substrate. Fluorescence signals were measured every 15 min for a total run of 50 hours. The *x*-axis represents the reaction time (hours), the *y*-axis represents the relative fluorescence units, and each curve represents a different dilution. Mean values of four replicates were used for each dilution. The cut-off (threshold) was based on the average fluorescence values of negative control + 5 × SD used in every assay.

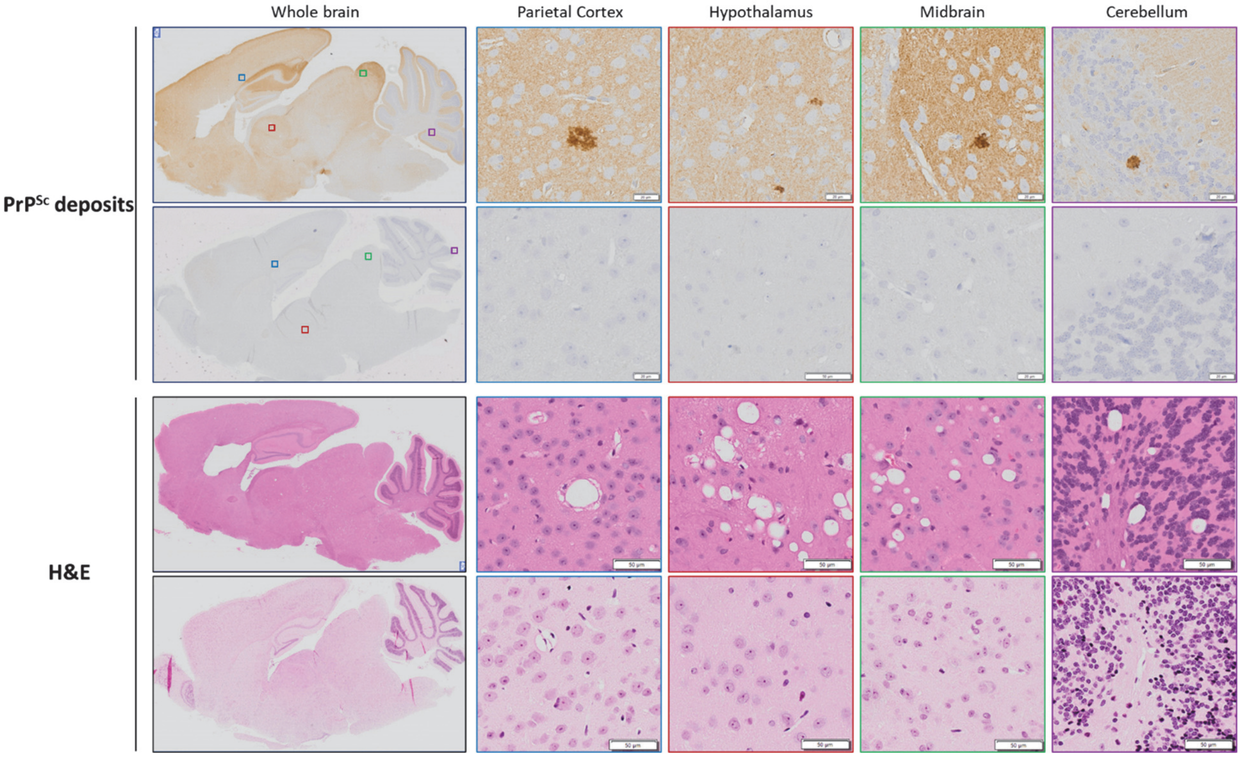

fig S20. Immunohistochemical and H&E analysis of brain tissue from bank vole #2583, inoculated with first passage TgElk-CWD-macaque.

Bank vole #2583 was inoculated with spinal cord homogenates of first passage TgElk-CWD macaque prions. IHC staining using SAF84 mAb, reveals PrP^{Sc} deposits in parietal cortex, hypothalamus, midbrain, and cerebellum brain regions with significant vacuolation seen in H&E staining (lower panels), absent in age-matched controls.

Scale bars 20µm (IHC) and 50 µm (H&E).

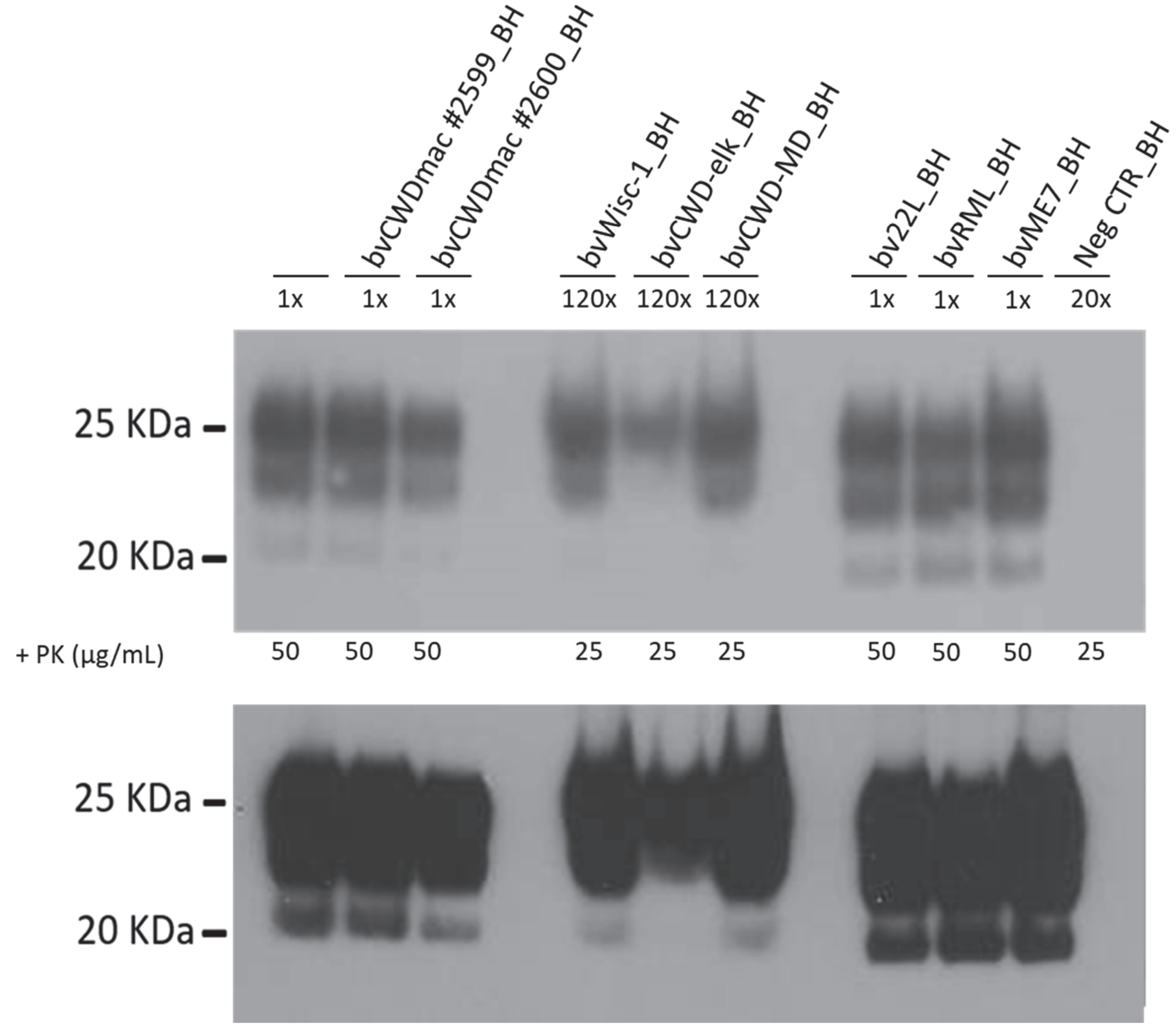

fig S21. Biochemical characterization of bvCWDmac compared to other prion strains to exclude potential contamination.

Brain homogenates from bvCWDmac and from scrapie adapted 22L, RML, and ME7 strains passaged in bank voles, were digested with 50 µg/mL of PK, while CWD isolates Wisc-1, elk, and mule deer passaged in bank voles were digested using 25 µg/mL of PK. We loaded 120 times more of the bvCWD material to have a comparable signal to bvCWDmac and scrapie adapted strains in bank voles. A negative control was also digested using 25 µg/mL of PK and loaded 20 times more compared to the volume loaded for bvCWDmac. **Lower panel is a longer exposure to show faint unglycosylated bands in bvCWD isolates.**

Animal No.	Route of inoculation	Inoculum	Years post inoculation	Clinical presentation	Clinical pathology
**AU242**	steel wire implantation (i.c.)	mock control material	5.67	Wasting	Hyperglycemia
**AU519**	steel wire implantation (i.c.)	CWD pool from WTD	5.16	– (scheduled PM)	–
**AU389**	steel wire implantation (i.c.)	CWD from elk	4.48	Anxiety<br>Ataxia<br>Tremor<br>Wasting	Hyperglycemia
**AU408**	intracerebral	10 mg CWD pool from WTD	6.56	–	Hyperglycemia
**AU469**	intracerebral	10 mg CWD pool from WTD	6.92	Wasting	Hyperglycemia
**AU467**	oral	5 x 2 g CWD pool	5.85	Wasting (died p.anest.)	–
**AU316**	oral	~5 kg CWD-muscle tissue (repeatedly)	7.49	Abdominal abscess	–
**AU501**	oral	~5 kg CWD-muscle tissue (repeatedly)	5.38	Anxiety<br>Ataxia<br>Tremor<br>Wasting	–

table S1. CWD-infected and control cynomolgus macaques.

Animal No.	Brain samples in which PrP^{Sc} was detected	Spleen samples in which PrP^{Sc} was detected	Route of inoculation
**AU242**	No	No	steel wire implantation (i.c.)
**AU519**	Yes	Yes	steel wire implantation (i.c.)
**AU389**	Yes	Yes	steel wire implantation (i.c.)
**AU408**	Yes	No	intracerebral
**AU469**	Yes	Yes	intracerebral
**AU467**	Yes	No	oral
**AU316**	No	ND	oral
**AU501**	Yes	Yes	oral
**DPZ16825**	No	No	
**DPZ16828**	No	No	
**Cov A – G**	No	No	
**BSE (A4)**	Yes	ND	intracerebral
**vCJD (+)**	Yes	Yes	
**No/Total No (positive macques tested)**	6/7	4/6	

table S2. Detection of PrP^{Sc} in macaque samples by PMCA Assay.
Macaques used to calculate the No/Total No (positive macaques tested): AU316, AU389, AU408, AU467, AU469, AU501, and AU519

Animal ID	Material	Passage	TgElk	dpi
AU501	Medulla	1st	0/5	
	Pons		0/5	
	Cerv. SC		1/5	182
	Thor. SC		0/10	
	Spleen		1/5	178*
AU519	Medulla	1st	1/5	150
	Pons		0/5	
	Cerv. SC		1/5	**344* (#2091)**
	Thor. SC		0/10	
	Spleen		0/10	
AU389	Pons	1st	0/5	
	Cerv. SC		0/10	
	Thor. SC		0/5	
	Spleen		1/5	94
AU467	Medulla	1st	0/5	
	Pons		0/5	
	Cerv. SC		1/5	206
	Thor. SC		1/5	171
	Spleen		0/5	
[a]AU242	Pons	1st	0/5	
[b]DPZ28	Medulla	1st	0/5	
DPZ28	Cerv. SC	1st	0/5	
TgElk AU501*	Spleen	2nd	1/5	134
TgElk AU519*	Cerv. SC	2nd	1/5	**106** (#2249)**

Suppl. Table 3. Transmission of CWD-macaque prions to transgenic mice expressing cervid PrP.
